# RdmA Is a Key Regulator in Autoinduction of DSF Quorum Quenching in Pseudomonas nitroreducens HS-18

**DOI:** 10.1128/mbio.03010-22

**Published:** 2022-12-20

**Authors:** Huishan Wang, Lingling Dong, Wenting Wu, Haowei Hu, Lian-Hui Zhang, Lisheng Liao

**Affiliations:** a Guangdong Province Key Laboratory of Microbial Signals and Disease Control, Integrative Microbiology Research Centre, South China Agricultural University, Guangzhou, China; b Guangdong Laboratory for Lingnan Modern Agriculture, Guangzhou, China; University of South Carolina; University of Hawaii at Manoa

**Keywords:** DSF, quorum quenching, autoinduction, transcriptional repressor, regulatory mechanism

## Abstract

Diffusible signal factor (DSF) represents a family of widely conserved quorum-sensing (QS) signals which regulate virulence factor production and pathogenicity in numerous Gram-negative bacterial pathogens. We recently reported the identification of a highly potent DSF-quenching bacterial isolate, Pseudomonas nitroreducens HS-18, which contains an operon with four DSF-inducible genes, *digABCD*, or *digA–D*, that are responsible for degradation of DSF signals. However, the regulatory mechanisms that govern the *digA–D* response to DSF induction have not yet been characterized. In this study, we identified a novel transcriptional regulator we designated RdmA (regulator of DSF metabolism) which negatively regulates the expression of *digA–D* and represses DSF degradation. In addition, we found that a gene cluster located adjacent to *rdmA* was also negatively regulated by RdmA and played a key role in DSF degradation; this cluster was hence named *dmg* (DSF metabolism genes). An electrophoretic mobility shift assay and genetic analysis showed that RdmA represses the transcriptional expression of the *dmg* genes in a direct manner. Further studies demonstrated that DSF acts as an antagonist and binds to RdmA, which abrogates RdmA binding to the target promoter and its suppression on transcriptional expression of the *dmg* genes. Taken together, the results from this study have unveiled a central regulator and a gene cluster associated with the autoinduction of DSF degradation in *P. nitroreducens* HS-18, and this will aid in the understanding of the genetic basis and regulatory mechanisms that govern the quorum-quenching activity of this potent biocontrol agent.

## INTRODUCTION

Quorum sensing (QS) is a widely conserved cell-cell communication mechanism by which bacteria synchronize and coordinate social activities such as pathogenesis and biofilm formation in a cell density-dependent manner. Many Gram-negative bacterial pathogens use either acyl-homoserine lactone (AHL) or diffusible signal factor (DSF) family molecules or both as QS signals in cell-cell communication. Among them, AHL-type QS systems have been well characterized since identification of the first AHL signal in the early 1980s (1), whereas the DSF-type QS system is relatively less characterized, as the chemical structure of DSF became known at a much later stage (2). The AHL QS systems consist of a LuxI-type enzyme responsible for synthesis of AHL signals and a LuxR-type protein that acts as a signal receptor and transcriptional regulator. Upon reaching a threshold level along with bacterial proliferation, AHL signal interacts with its cognate receptor and forms an AHL-LuxR complex, which induces transcriptional expression of the downstream virulence genes (3). Similarly, the DSF QS systems contain an RpfF-type signal synthase but rely on a two-component system for DSF signal detection and transduction. Typically in Xanthomonas campestris pv. *campestris*, at the pre-QS growth phase of low population density, the DSF sensor RpfC forms a complex with RpfF and thus limits DSF biosynthesis at a basal level, while at the QS phase of high population density, the accumulated extracellular DSF signals induce conformational changes in RpfC that initiate autophosphorylation and a phosphorelay to its cognate response regulator RpfG and release of RpfF, resulting in boosted DSF production and inducing expression of a few hundred genes associated with bacterial physiology and virulence (4–6).

Given the critical role of QS systems in modulation of bacterial pathogenesis, the quorum-quenching (QQ) strategy that aims to block QS through inactivation of QS signal or inhibition of signal perception has been proposed and proven to be a promising disease control alternative against QS-dependent pathogens (7–11). While most QQ studies have been performed with the well-characterized AHL QS systems, progress has also been made in identification of DSF degradation enzymes or counteracting mechanisms (12–15). In our previous study, a bacterial isolate of Pseudomonas nitroreducens HS-18 with superior QQ capacity against DSF signals was identified by using a highly efficient and reliable screening method. Genetic analysis unveiled an operon containing 4 *fadD* homologues (*digABCD*, or *digA–D*) responsible for DSF degradation and the biocontrol potency of strain HS-18 (15, 16). Intriguingly, expression levels of *digA–D* were all upregulated in the presence of DSF (15), suggesting that the transcriptional expression of these DSF QQ genes was under the control of an unknown regulatory mechanism(s) that could receive and respond to exogenous DSF signals.

DSF family signals are the *cis*-2-unsaturated fatty acid derivatives, which differ in chain length and methyl substitutions. Fatty acid metabolism and regulation have been well studied in Escherichia coli. In E. coli, fatty acid degradation is catalyzed by the enzymes encoded by seven *fad* genes, including *fadA*, *fadB*, *fadD*, *fadE*, *fadI*, *fadJ*, and *fadL*, via β-oxidation. Fatty acid molecules are assimilated by the outer membrane protein FadL and activated by the inner membrane-associated fatty acid-coenzyme A (CoA) ligase FadD, and the acyl-CoA dehydrogenase FadE is responsible for the first step of conversion of acyl-CoA to enoyl-CoA; the enoyl-CoA hydratase FadB and acetyl-CoA C-acyltransferase FadA are responsible for hydration and oxidation and shortening, respectively, of acyl-CoA (17). Unsaturated fatty acid degradation needs 2,4-dienoyl-CoA reductase FadH for transforming unsaturated fatty acids to enoyl-CoA (18). Transcriptional expression of the *fad* genes is under the control of FadR, which is a member of the GntR family and acts as a negative regulator (18, 19). Recently, RpfB of the plant bacterial pathogen Xanthomonas campestris pv. *campestris*, which shows a high level of homology to the FadD of E. coli, was found to play a key role in enzymatic turnover of DSF signal molecules. However, X. campestris pv. *campestris* doesn’t seem to encode a FadR homologue, and *rpfB* expression is controlled by Clp, which is a c-di-GMP effector that belongs to the CRP-FNR family (13, 20). These findings suggest that the fatty acid degradation pathway may be conserved but that the corresponding regulatory mechanisms could be different.

In this study, to understand how DSF induces its degradation in the biocontrol agent *P. nitroreducens* HS-18, we initially considered the possible involvement of FadR and Clp homologues in regulation of DSF quenching through modulation of *digA–D* transcriptional expression. Bioinformatics analysis showed that strain HS-18 encodes 9 FadR and 7 Clp homologues; however, the results of our preliminary genetic analysis didn’t seem to support their roles in regulation of *digA–D* (data not shown). We therefore conducted transcriptome analysis to screen for the potential regulatory genes influenced by exogenous addition of DSF, which led to identification of the transcriptional regulator RdmA, belonging to the TetR family. Subsequent analysis showed that in a DSF-dependent manner, RdmA modulates the transcriptional expression of *digA–D* and a new gene cluster associated with DSF metabolism (*dmgABCDEFGH*), respectively. These findings shed new light on DSF degradation and regulatory mechanisms, which may serve as useful clues for further improving the biocontrol efficiency of Pseudomonas sp. strain HS-18.

## RESULTS

### Identification of potential regulators modulating DSF degradation activity of *P. nitroreducens* HS-18.

**FIG 1:**
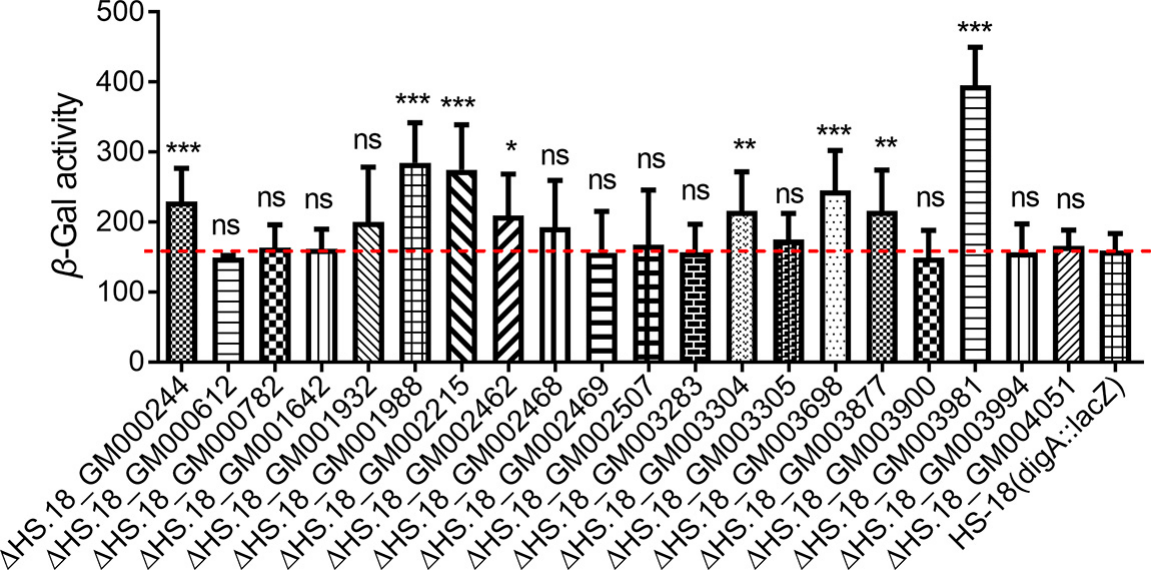
Identification of potential transcription factors affecting *digA* expression in Pseudomonas sp. strain HS-18. The differentially expressed regulatory genes with or without DSF (see Table S2 in the supplemental material) were deleted using the strain HS-18(P*_digA_*-*lacZ*), in which the reporter gene *lacZ* was placed under the control of the *digA* promoter (P*_digA_*), as the parental strain. The reporter and mutants were grown separately in MM* medium supplemented with 0.5 mM DSF for 6 h, and the β-galactosidase activities were measured. The gene *HS.18_GM003981*, which showed the most significant effect on the promoter activity of *digA*, was then designated *rdmA* (regulator of DSF metabolism). Statistical analyses were performed using the *t* test and two-way analysis of variance (ANOVA). The experiment was repeated at least 3 times with triplicates. Bars indicate the means with standard deviations (SD) of three independent repeats. *, *P* < 0.05; **, *P* < 0.01; ***, *P* < 0.001; ns, not significant.

Our previous study showed that the *digA–D* genes were significantly upregulated in the presence of DSF QS signals (15). These findings implied that transcriptional expression of certain regulatory genes governing DSF metabolism may also be changed by DSF. Following this speculation, we conducted transcriptome analysis of strain HS-18 cultured in the presence and absence of DSF with an aim to identify DSF-inducible regulatory genes. The results revealed that there were about 20 regulatory genes that showed significant changes at the transcript level in the presence of DSF. Among these genes, 16 were upregulated and 4 genes were downregulated (see Table S2 in the supplemental material). To determine whether these regulators play a role in regulation of DSF degradation, we constructed the corresponding deletion mutants using *P. nitroreducens* HS-18 (*digA*::*lacZ*) as the parental strain, and the β-galactosidase activities of the mutants and their parental strain were measured in the presence of DSF. Among the 20 mutants assayed in this study, 8 of them showed significantly changed expression of *digA* in comparison with the parental strain HS-18(*digA::lacZ*) (Table S2 and Fig. 1). In particular, deletion of *HS.18_GM003981* resulted in the most significantly upregulated *digA* expression. We hence named *HS.18_GM003981 rdmA* (regulator of DSF metabolism) and used it for further characterizations in this study.

10.1128/mbio.03010-22.2 TABLE S2 Differentially expressed regulatory genes in the presence and absence of DSF. Download Table S2, DOCX file, 0.02 MB. Copyright © 2022 Wang et al. 2022 Wang et al. https://creativecommons.org/licenses/by/4.0/ This content is distributed under the terms of the Creative Commons Attribution 4.0 International license.

Bioinformatics analysis showed that *rdmA* encodes a peptide of 206 amino acids, and its product, RdmA, contains a DNA-binding domain with helix-turn-helix structure in its N terminus, belonging to the TetR-like transcription factor family. RdmA showed a low similarity to FadR (<10%) and no similarity to Clp, which are the known repressors of fatty acid metabolism and DSF turnover in E. coli and X. campestris pv. *campestris*, respectively (13, 21), but it presented a high similarity (86.29%) to AtuR, which is the repressor of the *atu* (acyclic terpene utilization) gene cluster in Pseudomonas aeruginosa and Pseudomonas citronellolis (22).

### The null mutation of RdmA in strain HS-18 resulted in enhanced DSF degradation activity.

**FIG 2:**
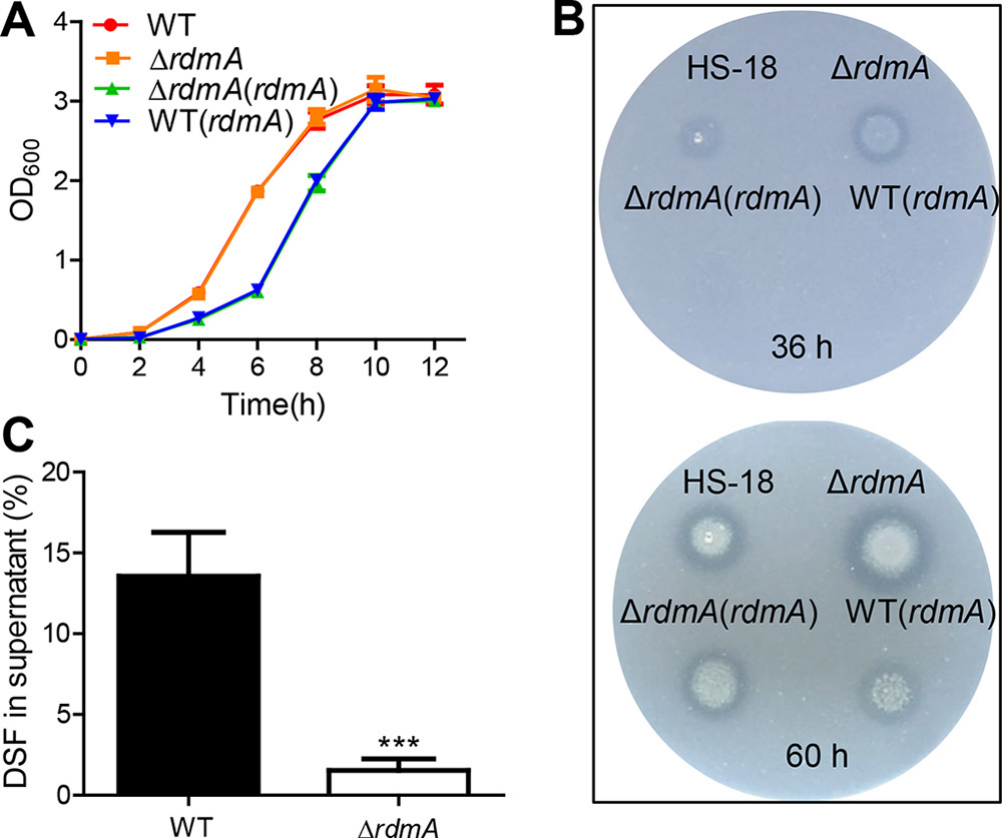
Effect of *rdmA* on DSF degradation capacity of strain HS-18. (A) Effect of *rdmA* deletion or overexpression on bacterial growth in LB medium. (B) DSF degradation zones produced by WT HS-18 and the *rdmA* deletion mutants on the MM* plate with 5 mM DSF as sole carbon source. (C) HPLC quantitative analysis of the remaining DSF in the culture supernatants of strain HS-18 and the mutant Δ*rdmA* in MM* liquid medium with 0.5 mM DSF as the sole carbon source at 9 h postincubation. The experiment was repeated at least 3 times with triplicates. Statistical analyses were performed using the *t* test and two-way ANOVA. Bars indicate means with SD of three independent repeats. ***, *P* < 0.001.

To determine the role of RdmA in DSF degradation, we constructed the *rdmA*-disrupted mutant, Δ*rdmA*, using wild-type (WT) HS-18 as the parental strain, the correspondingly complementary strain Δ*rdmA*(*rdmA*), and the *rdmA* overexpression strain WT(*rdmA*). The results showed that strain HS-18 and its *rdmA* deletion mutant resulted in no significant differences in growth in modified minimal medium (MM*) containing 10 mM succinate as a carbon source, with or without DSF (Fig. S1) or in LB medium (Fig. 2A), but the complemented strain and overexpression strain showed delayed logarithmic growth compared to strains HS-18 and mutant Δ*rdmA* (Fig. 2A and Fig. S1).

10.1128/mbio.03010-22.5 FIG S1 Growth curves of strain HS-18, the *rdmA* deletion mutant, and the complemented strain. The bacterial cells were inoculated in MM* medium with addition of 10 mM succinate (A) or in MM* medium with addition of 10 mM succinate and 0.5 mM DSF (B). Error bars represent SD, calculated from three independent assays. Download FIG S1, TIF file, 0.9 MB. Copyright © 2022 Wang et al. 2022 Wang et al. https://creativecommons.org/licenses/by/4.0/ This content is distributed under the terms of the Creative Commons Attribution 4.0 International license.

DSF degradation activities of strain HS-18 and its derivatives were evaluated using both a DSF plate assay and high-performance liquid chromatography (HPLC) quantitative analysis, as described previously (15). The results showed that the degradation zone produced by Δ*rdmA* was obviously bigger than the one produced by strain HS-18 on the MM* plate with 5 mM DSF as sole carbon source at either 36 h or 60 h postinoculation (Fig. 2B), suggesting that RdmA could act as a repressor on DSF quenching in strain HS-18. In *trans* expression of *rdmA* in the deletion mutant Δ*rdmA* restored DSF degradation to a level comparable to that of strain HS-18 at 60 h postinoculation. Consistent with the notion that RdmA is a transcriptional repressor, the *rdmA* overexpression strain WT(*rdmA*) produced a smaller degradation zone than the complemented strain Δ*rdmA*(*rdmA*) or wild-type HS-18 (Fig. 2B). To quantitatively assess the DSF degradation activity of strain HS-18 and mutant Δ*rdmA*, bacterial cells were added to the MM* liquid medium containing 5 mM DSF as sole carbon source, and the residual DSF molecules in the culture supernatants were analyzed by HPLC at 9 h after inoculation. In agreement with the findings of the plate assay, the quantitative results showed that the mutant Δ*rdmA* degraded DSF at a much faster rate than strain HS-18 (Fig. 2C).

### RdmA acts as a repressor in regulation of DSF degradation genes *digABCD*.

**FIG 3:**
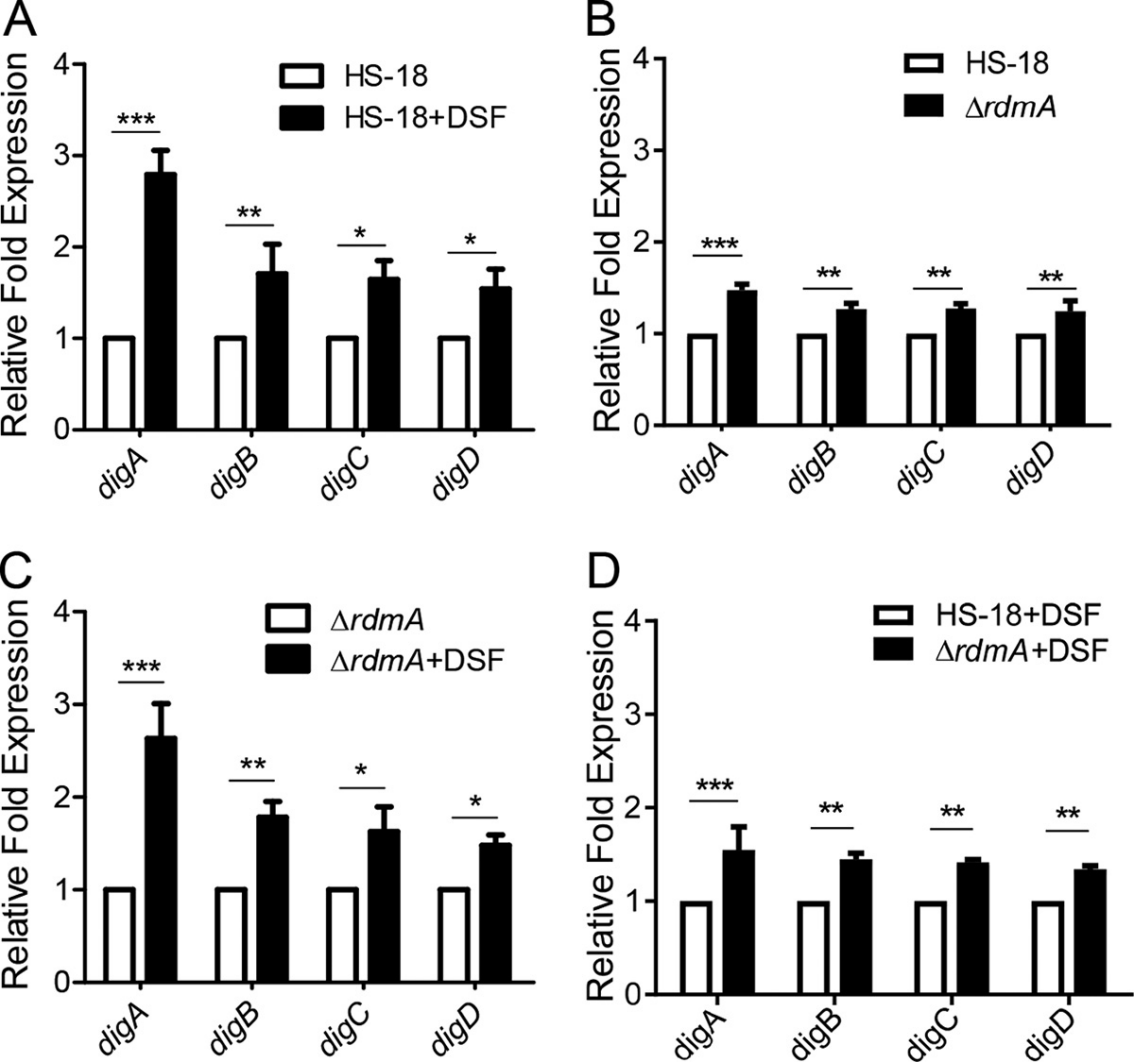
Effect of *rdmA* on transcriptional expression of the DSF-degrading genes *digA*–*digD*. (A and D) Relative expression levels of *digA–-digD* were quantified by RT-qPCR using RNA extracted from HS-18 (A) or Δ*rdmA* (C) grown in MM* liquid medium containing 10 mM succinate (basic medium) in the absence or presence of 0.5 mM DSF, respectively, at 5 h postinoculation. (B and D) Relative expression levels of *digA–digD* between HS-18 and Δ*rdmA* grown in basic medium in the absence (B) or presence (D) of 0.5 mM DSF, respectively, at 5 h postinoculation were also quantified by RT-qPCR using extracted RNA. All data were normalized to the transcript level of the housekeeping gene *rpoD*. The experiment was repeated at least 3 times with triplicates. Statistical analyses were performed using the *t* test and two-way ANOVA. Bars indicate the means with SD of three independent repeats. *, *P* < 0.05; **, *P* < 0.01; ***, *P* < 0.001.

Deletion of *rdmA* in the reporter strain HS-18 (*digA*::*lacZ*) and in wild-type HS-18 resulted in significantly increased LacZ enzyme activity (Fig. 1) and boosted DSF degradation activity (Fig. 2), respectively, which strongly suggested that RdmA is a negative regulator of *digA* expression. To validate this notion, the effect of *rdmA* on regulation of the transcriptional expression of *digA–D* in strain HS-18 was examined by reverse transcription-quantitative PCR (RT-qPCR). The results showed that addition of DSF increased the transcriptional levels of *digA–D* in either strain HS-18 or the mutant Δ*rdmA* (Fig. 3A and C), and this suggested that DSF might act in induction of *dig* expression by counteracting the inhibitory activity of RdmA. In agreement with this speculation, deletion of *rdmA* led to enhanced expression of the *dig* genes, compared to strain HS-18 in either the presence or absence of DSF (Fig. 3B and D). These findings indicated that RdmA is a negative regulator on transcriptional expression of the *dig* genes, which were shown previously to encode DSF degradation enzymes (15).

### The *dmgABCDEFGH* gene cluster adjacent to *rdmA* is involved in DSF metabolism.

Genome analysis showed that the *rdmA* gene is located adjacent to a gene cluster consisting of *HS.18_GM003982* to *HS.18_GM003989* (here designated *dmgA–H*) in the opposite transcriptional direction in strain HS-18 (Fig. S2A). Transcriptome analysis revealed that both *rdmA* and the *dmgA–-H* gene cluster were significantly upregulated in the presence of DSF, with the exception of *dmgH*, which encodes a homologue of DSF degradation enzyme DigA–D (15) (Table S3). BLAST analyses revealed that RdmA and DmgA–H showed high similarities (79.06 to 91.08%) to AtuR and AtuA–H, respectively, in P. aeruginosa (Table S3). The *atu* gene cluster was previously identified to be essential for utilization of acyclic terpene in P. aeruginosa and P. citronellolis (22–24). DSF family signal molecules share certain similar structural features as acyclic terpenes (22, 25), which suggests that the predicted products of the *dmg* gene cluster are likely associated with DSF degradation (Table S3).

10.1128/mbio.03010-22.3 TABLE S3 Similarities of proteins encoded by the *dmg* cluster in strain HS-18 with the corresponding proteins in P. aeruginosa PAO1. Download Table S3, DOCX file, 0.02 MB. Copyright © 2022 Wang et al. 2022 Wang et al. https://creativecommons.org/licenses/by/4.0/ This content is distributed under the terms of the Creative Commons Attribution 4.0 International license.

10.1128/mbio.03010-22.6 FIG S2 Genetic organization and transcription unit analysis of the *dmg* cluster. (A) Schematic diagram of the *rdmA* and *dmg* gene clusters of *P. nitroreducens* HS-18. The intergenic regions and the lengths between *rdmA*-*dmgA* (lane 1) and the neighboring dmg genes (lanes 2 to 8) are indicated. (B) RT-PCR analysis of the transcription units of the *dmg* gene cluster. The cDNA samples from *P. nitroreducens* HS-18 grown in the presence (+ lanes) or absence (− lanes) of DSF were used as the template to amplify the intergenic regions 1 to 8. The genomic DNA (lane g) was used as a control to amplify the corresponding intergenic regions with the same primers. Download FIG S2, TIF file, 1.5 MB. Copyright © 2022 Wang et al. 2022 Wang et al. https://creativecommons.org/licenses/by/4.0/ This content is distributed under the terms of the Creative Commons Attribution 4.0 International license.

To explore the role of the *dmg* cluster in DSF metabolism, we first tested the transcribed transcript of genes in the *dmg* cluster with or without DSF. The intergenic regions of *rdmA* and the *dmg* cluster were analyzed by RT-PCR using the primers listed in Table S1B. The genomic DNA of strain HS-18 was taken as a positive control. The results showed that the intergenic regions of *dmgA*-*dmgB*, *dmgB*-*dmgC*, *dmgC*-*dmgD*, *dmgE*-*dmgF*, and *dmgF*-*dmgG* were successfully amplified using the cDNA of strain HS-18 cultured in the presence of DSF, whereas the intergenic regions of *rdmA*-*dmgA*, *dmgD*-*E*, and *dmgG*-*H* could not be detected (Fig. S2B). These findings thus indicated that the presence of DSF had a significant effect on transcription of the *dmg* cluster.

10.1128/mbio.03010-22.1 TABLE S1 (A) Bacterial strains used in this study. (B) Primers used in this study. Download Table S1, DOCX file, 0.04 MB. Copyright © 2022 Wang et al. 2022 Wang et al. https://creativecommons.org/licenses/by/4.0/ This content is distributed under the terms of the Creative Commons Attribution 4.0 International license.

**FIG 4:**
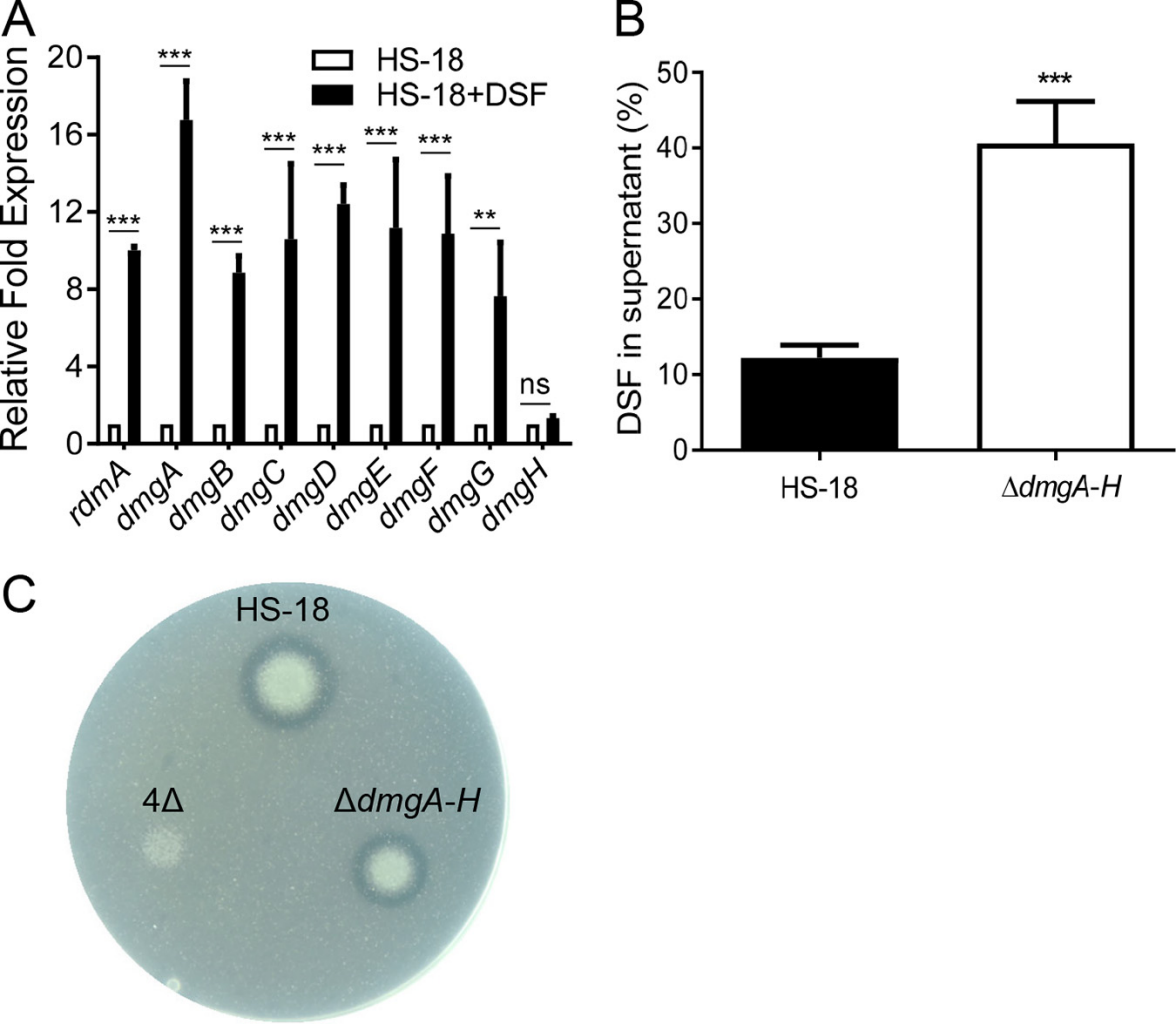
The *dmg* gene cluster is associated with DSF metabolism. (A) DSF boosted transcriptional expression of *rdmA*–*dmgH* in strain HS-18, which was cultured in MM* medium containing 10 mM succinate with or without 0.5 mM DSF. The transcript levels at 6 h postinoculation were determined by using RT-qPCR. All the data were normalized to the *rpoD* value. (B) Deletion of the *dmg* cluster compromised the DSF degradation activity of strain HS-18. Wild type HS-18 and its mutant, Δ*dmgA*–*H*, were cultured in MM* medium with 0.5 mM DSF as sole carbon source for 9 h. The remaining DSF signals in the culture supernatants were measured by HPLC. (C) DSF degradation zones produced by WT HS-18, the *digA*–*D* deletion mutant 4Δ, and Δ*dmgA*–*H* on MM* plates with 5 mM DSF as sole carbon source. The photo was taken at 60 h postincubation. The experiment was repeated at least 3 times with triplicates. Statistical analyses were performed using the *t* test and two-way ANOVA. Bars indicate the means with SD of three independent repeats. **, *P* < 0.01; ***, *P* < 0.001; ns, not significant.

To further confirm the involvement of DSF in the regulation of the *dmg* expression, transcriptional analysis of *rdmA* and *dmgA–H* was performed by RT-qPCR in the presence and absence of DSF, respectively. The results showed that the transcript levels of *rdmA* and all the *dmg* genes except *dmgH* were significantly increased in strain HS-18 treated with DSF compared to the control without DSF (Fig. 4A). The data were highly consistent with the results of the transcriptome analysis, which defined all the *dmg* genes except *dmgH* as DSF-inducible genes (Table S3). The above findings therefore demonstrated unequivocally that transcriptional expression of the *rdmA* and *dmgA–G* genes is mediated by DSF signals.

To assess the role of Dmg enzymes in DSF degradation, we generated the mutant Δ*dmgA*–*H* by deleting all the genes in the *dmgA*–*H* cluster using wild-type HS-18 as the parental strain. The results (Fig. 4B) showed that deficiency of *dmgA*–*H* significantly impaired the DSF degradation capacity of strain HS-18, suggesting that the *dmg* cluster is involved in DSF metabolism. We further compared the impact of *dmg* deletion with that of *dig* deletion on DSF degradation in a plate assay. The results showed the *dig* deletion compromised more of the DSF degradation capacity of strain HS-18 than did the *dmg* deletion (Fig. 4C).

### RdmA is a negative regulator modulating transcriptional expression of *dmgA–G*.

**FIG 5:**
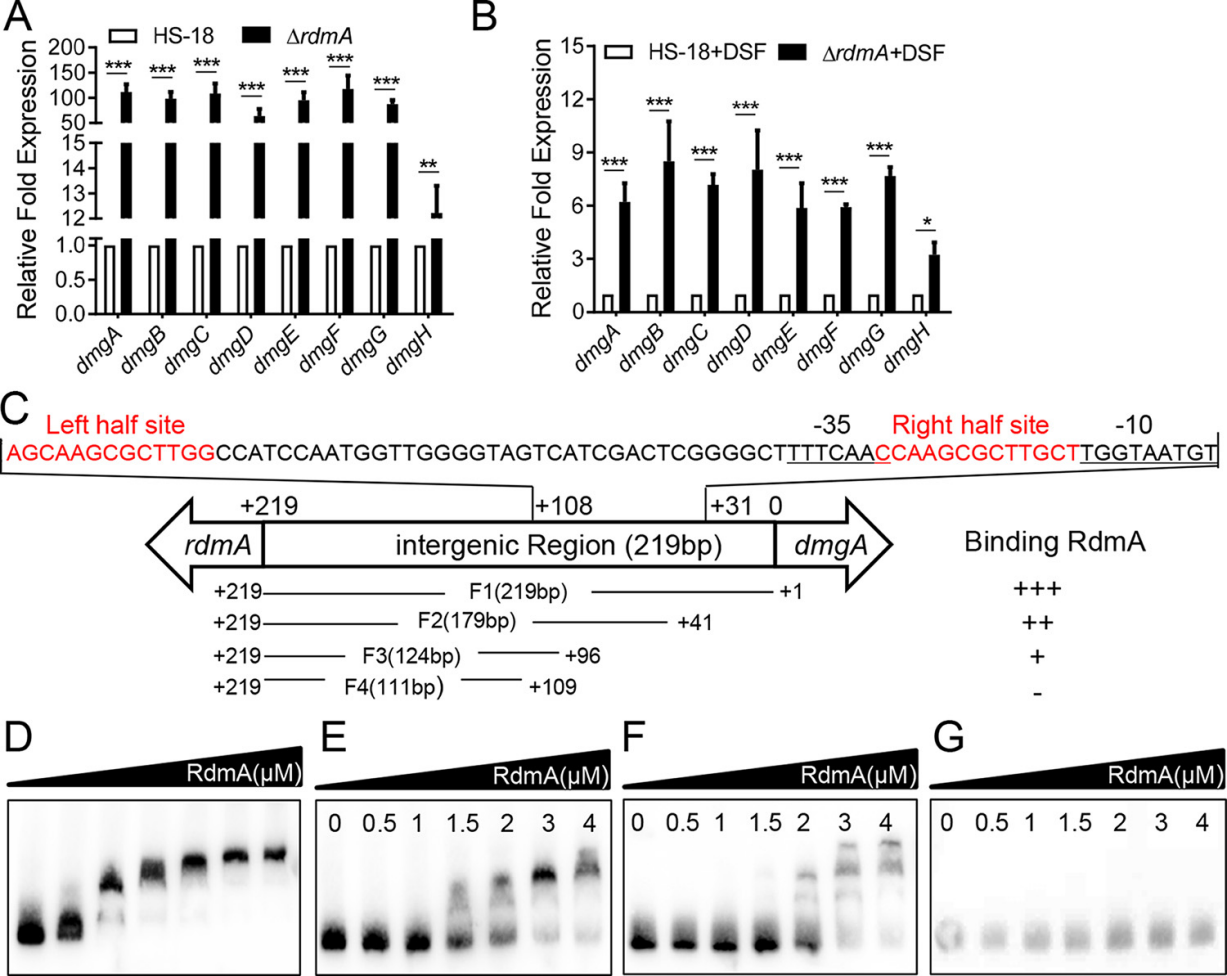
Molecular mechanism of RdmA in modulation of *dmg* transcription. (A and B) Relative transcriptional levels of *dmgA*–*dmgH* in strain HS-18 and mutant Δ*rdmA* in MM* medium containing 10 mM succinate with (A) or without (B) 0.5 mM DSF. The transcript levels at 6 h postinoculation were determined by using RT-qPCR, and the data were normalized to the *rpoD* value. (C) Schematic diagram of the *rdmA*-*dmgA* intergenic region and DNA fragments used to determine the *rdmA* binding site. The position 0 indicates the ATG start codon of *dmgA*. The sequence at the top shows the region from position +31 to +108. Red sequences represent the two inverted repeated sequences (IRSs). The −10 and −35 boxes are indicated. Fragment 1 (F1) represents the whole intergenic region of *rdmA* and *dmgA* from +1 to +219. Fragment 2 (F2) indicates the sequence from position +41 to +219, which contains the two IRSs. Fragment 3 (F3) indicates the sequence from position +96 to +219, which contains only the left half of the IRS. Fragment 4 (F4) indicates the sequence from position +109 to +219, which contains no IRS. (D, E, F, and G) EMSA of RdmA (0 to 4 μM) binding to fragments F1 to F4 (10 nM), respectively. The experiment was repeated at least 3 times with triplicates. Statistical analyses were performed using the *t* test and two-way ANOVA. Bars indicate the means with SD of three independent repeats. *, *P* < 0.05; **, *P* < 0.01; ***, *P* < 0.001.

The adjacent locations of *rdmA* and the *dmg* gene cluster suggest that the regulator RdmA might be associated with the modulation of *dmg* expression. Towards this end, we compared the transcriptional expression levels of the *dmg* genes in wild-type HS-18 and the mutant Δ*rdmA* by RT-qPCR. The results showed that deletion of *rdmA* led to drastic increases in the transcript levels of all the *dmg* genes, especially *dmgA–H*, in comparison with their expression levels in the background of wild-type strain HS-18 (Fig. 5A). We then tested the influence of *rdmA* deletion on transcriptional expression of the *dmg* gene cluster in the presence of DSF. Interestingly, compared with the >70-fold differences in transcript levels for most of the *dmg* genes in strain HS-18 and mutant Δ*rdmA* without DSF (Fig. 5A), the impact of *rdmA* deletion on *dmg* gene expression became much lower in the presence of DSF, in the range of 3- to 9-fold (Fig. 5B). Taken together, the findings suggest that, similar to its regulatory role in expression of the *digABCD* genes (Fig. 3B and D), RdmA is a negative transcriptional regulator in modulation of the *dmg* gene cluster. However, RdmA may not be the only transcription factor that controls the expression of the *dmg* gene cluster.

### RdmA directly binds to two inverted repeat sequences in the promoter of *dmgA*.

Bioinformatics analysis of RdmA showed that RdmA belongs to the TetR family of regulators, which are known as ligand-bound and DNA-bound transcriptional regulators (26). TetR-like proteins usually act as repressors via binding in the form of dimers to the promoter regions of target genes (27, 28). To investigate the potential association of RdmA with the *dmg* cluster, the promoter region of *dmgA* was predicted using the online Softberry software BPROM program (https://www.softberry.com/berry.phtml?topic=bprom&group=programs&subgroup=gfindb) (Fig. 5C). EMSA was conducted with purified His_6_-tagged RdmA and the 219-bp DNA fragment of the promoter region of *dmgA* (P*_dmgA_*) (fragment F1 in Fig. 5C). The results showed that RdmA directly bound to P*_dmgA_* in a concentration-dependent manner (Fig. 5D). RdmA was highly similar to the negative regulator AtuR of P. aeruginosa, with about 86.29% identity at the amino acid level. AtuR was characterized to directly bind to the promoter region of *atuA*, of which the two 13-bp inverted repeat sequences (IRSs; AGCAAGCGCTTGG and CCAAGCGCTTGCT), located immediately upstream of the −10 region and separated by a spacer sequence of 40 bp, were confirmed to be the AtuR-binding site (22). In strain HS-18, we also identified the two 13-bp IRSs in the promoter of *dmgA* which were identical to the IRS of the *atuA* promoter but separated by a spacer sequence of 42 bp (Fig. S5). To validate the above-identified potential binding site of RdmA on P*_dmgA_*, three promoter fragments of varied lengths were PCR amplified for EMSA analysis. Among them, fragment F2 was 179 bp and contained an intact left-half IRS and right-half IRS without sequences located downstream of the right-half IRS, fragment F3 was 124 bp without sequences located downstream of the left-half IRS, and fragment F4 was 111 bp with both right- and left-half IRSs being removed (Fig. 5C). The EMSA with different amounts of RdmA revealed that compared with the intact promoter (fragment F1), with which addition of 1 μM RdmA could cause complete mobility shift (Fig. 5D), promoter segments F2 and F3 required over 1.5 and 2 μM RdmA to result in partial motility shifts, respectively (Fig. 5E and F), whereas segment F4 was not able to interact with RdmA (Fig. 5G). Hence, the results certified that the two 13-bp IRSs of the *rdmA*-*dmgA* intergenic region were essential for physical interaction with RdmA.

10.1128/mbio.03010-22.9 FIG S5 Sequence alignment of the *dmgA* promoter and its homologues in other microorganisms. The two inverted repeat sequences that are vital for the binding of RdmA to *dmgA* are conserved in various Pseudomonas species and other genera. Right inverted repeat sequence CCAAGCGCTTGCT (red) is widely conserved, while left inverted repeat sequence CCAAGCGCTTGCT (blue) is less conserved. Highly conserved residues (100% identity) and partially conserved residues (75 to 100% identity) are shaded in black and gray, respectively. Download FIG S5, TIF file, 2.9 MB. Copyright © 2022 Wang et al. 2022 Wang et al. https://creativecommons.org/licenses/by/4.0/ This content is distributed under the terms of the Creative Commons Attribution 4.0 International license.

### DSF prevents RdmA binding to the *dmgA* promoter region.

**FIG 6:**
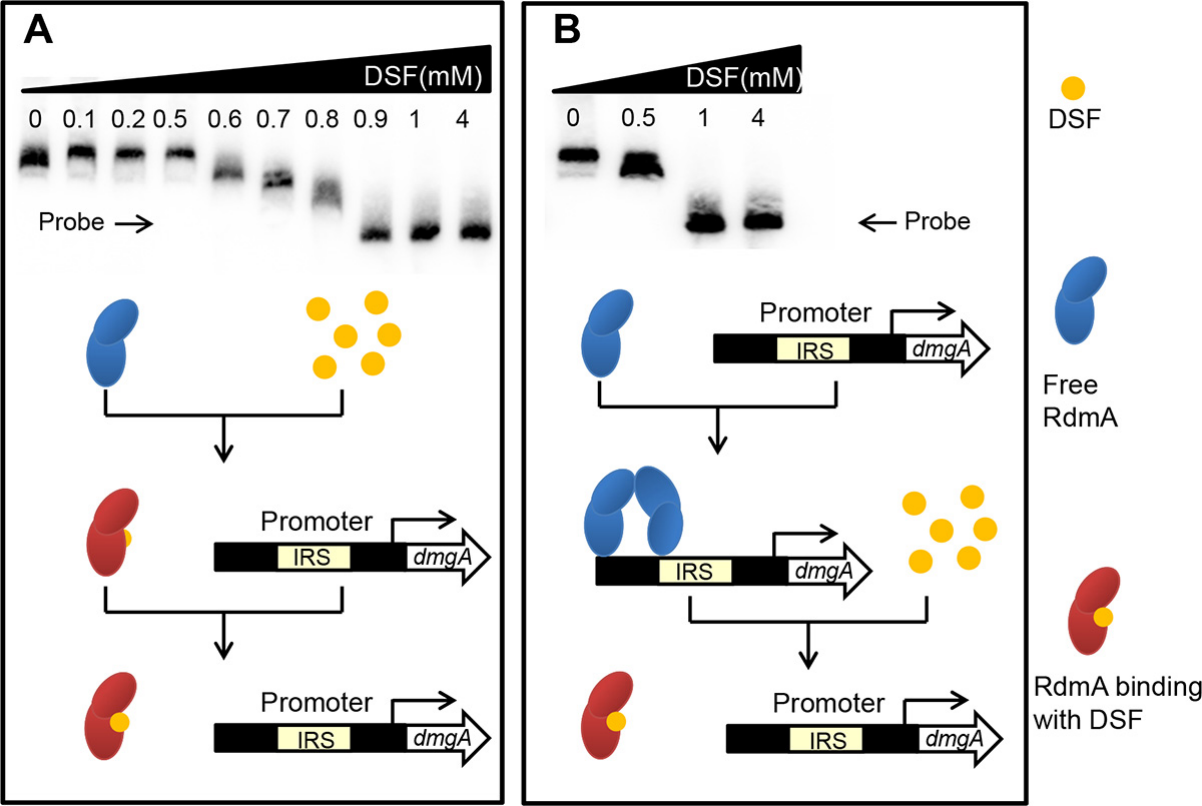
Effect of DSF on binding of RdmA to the promoter P*_dmgA_*. (A) EMSA analysis of the RdmA-P*_dmgA_* interaction with RdmA incubated with DSF for 30 min prior to addition of the promoter P*_dmgA_*. (B) EMSA analysis of the RdmA-P*_dmgA_* interaction with RdmA incubated with P*_dmgA_* for 30 min prior to addition of DSF. Purified RdmA and P*_dmgA_* were added at final concentrations of 2 μM and 20 fM, respectively, and the concentrations of DSF used ranged from 0 to 4 mM. The solvent methanol, which was used to dissolve DSF signals, was used as a negative control in each assay. The experiments were repeated at least 3 times with similar results.

The results in Fig. 5A and B show that DSF might counteract the repressor RdmA in regulation of DSF degradation. To examine this possibility, we tested the effect of DSF on the interaction of RdmA and P*_dmgA_* by EMSA using two approaches. We first tested the effect of DSF on RdmA by mixing the signal and regulator prior to addition of the *dmgA* promoter. The mixture of DSF and RdmA was incubated at 30°C for 30 min, and then the *dmgA* promoter (fragment F1 in Fig. 5C) labeled by biotin was added into the mixture, followed by incubation at 22°C for another 30 min. The EMSA results showed that addition of DSF at 0.6 mM led to decreased formation of the RdmA-P*_dmgA_* complex, and 0.9 mM DSF completely inhibited the binding of RdmA to P*_dmgA_* (Fig. 6A). In the second approach, we tested whether DSF could dissociate the preformed RdmA-P*_dmgA_* complex. The mixture of P*_dmgA_* DNA and RdmA was incubated first at 22°C for 30 min, and then DSF was added to the mixture for 30 min of incubation, again at 30°C. The EMSA results revealed that DSF was able to disrupt the RdmA-P*_dmgA_* complex, and complete dissociation was observed at 1 mM DSF (Fig. 6B). Therefore, DSF is a valid ligand in modulation of the functionality of the negative regulator RdmA and an effective inducer for initiating transcriptional expression of the *dmg* gene cluster.

### Ligand specificity of RdmA.

**FIG 7:**
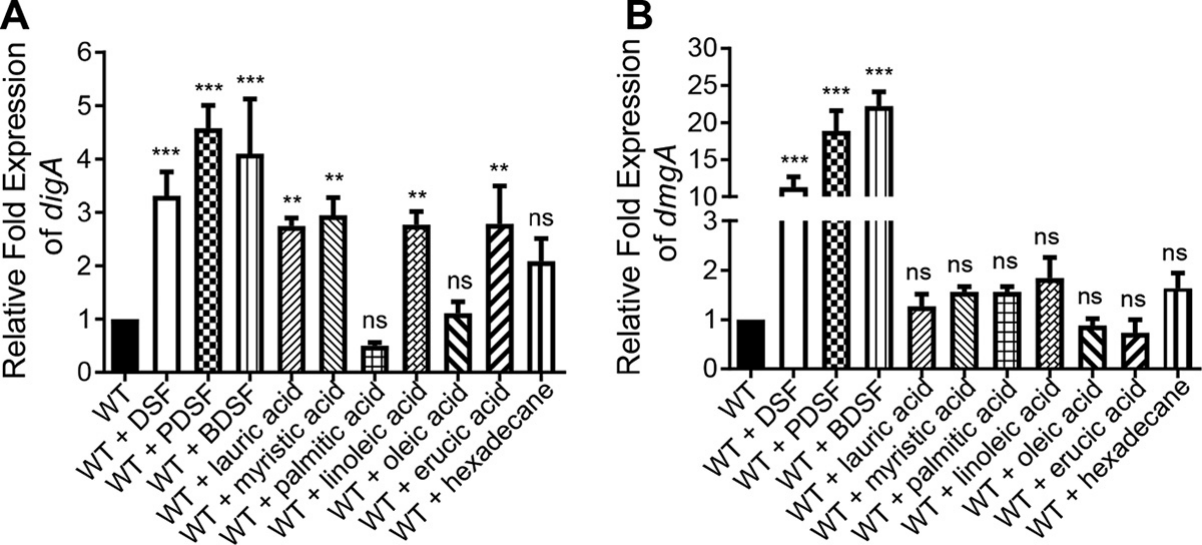
RT-qPCR analyses of transcript levels of *digA* and *dmgA* in the presence of different fatty acids. Data shown are the relative expression levels of *digA* (A) and *dmgA* (B) in WT HS-18 grown in MM* liquid medium containing 10 mM succinate in the presence of DSF or derivatives at a final concentration of 0.5 mM. The RNA samples were purified from the bacterial cultures at 5 h postinoculation. All data were normalized to the *rpoD* value. The experiments were repeated at least 3 times with triplicates. Statistical analyses were performed using the *t* test and two-way ANOVA. Bars indicate the means with SD of three independent repeats. *, *P* < 0.05; **, *P* < 0.01; ***, *P* < 0.001; ns, not significant.

To detect the ligand specificity of RdmA, a range of fatty acid molecules, including *cis*-2-decenoic acid (PDSF), *cis*-2-dodecenoic acid (BDSF), lauric acid, myristic acid, palmitic acid, linoleic acid, oleic acid, and erucic acid, were used to determine their effect on the functionality of RdmA (Table S4). We first confirmed the influence of these fatty acid molecules on the transcript levels of *digA* and *dmgA* in strain HS-18. RT-qPCR analysis showed that addition of DSF, PDSF, BDSF, lauric acid, myristic acid, linoleic acid, or erucic acid significantly upregulated the expression of *digA*, especially the 3 DSF family signals (Fig. 7A). Similarly, DSF, PDSF, and BDSF also presented a significantly upregulating influence on the transcript level of *dmgA* compared to other fatty acids (Fig. 7B).

10.1128/mbio.03010-22.4 TABLE S4 Chemical structures of DSF and fatty acid derivatives. Download Table S4, DOCX file, 0.1 MB. Copyright © 2022 Wang et al. 2022 Wang et al. https://creativecommons.org/licenses/by/4.0/ This content is distributed under the terms of the Creative Commons Attribution 4.0 International license.

## DISCUSSION

Genetic regulation of QS mechanisms have been well-studied in the last few decades (3, 4, 6), whereas how QQ is modulated remains largely unknown. In this study, we report the identification of a transcriptional regulator RdmA in *P. nitroreducens* HS-18, which plays a key role in modulation of the transcriptional expression of the 4 previously identified DSF-inducible genes encoding highly active DSF degradation enzymes (15). In addition, we unveiled a new gene cluster designated *dmgABCDEFGH* that is involved in DSF metabolism. Furthermore, we demonstrated that RdmA acts as a negative repressor on the transcriptional expression of *digABCD* and *dmgABCDEFGH*, and DSF could effectively abrogate this repression by interacting with RdmA. These original findings have thus added a new chapter in elucidating the molecular mechanisms with which microorganisms can modulate QQ activities.

Our previous study identified 4 *dig* genes encoding enzymes for DSF degradation (15). The products of these *dig* genes share high similarities to the FadB of E. coli, which is an enoyl-CoA ligase responsible for activation of fatty acid to produce fatty acyl-CoA (17). These findings suggested that DSF degradation may share a similar pathway as fatty acid metabolism. In E. coli, transcriptional expression of the *fad* genes is under the control of FadR, which is a member of the GntR family and acts as a negative regulator (17, 19). In this study, bioinformatics analysis unveiled at least 9 genes encoding FadR homologues; however, none of them appeared to be involved in DSF metabolism in *P. nitroreducens* strain HS-18. Instead, we found that a transcriptional regulator, RdmA, belonging to the TetR family plays a key role in regulation of *dig* gene expression and DSF metabolism. The best homologue of RdmA is AtuR, which is the repressor of the *atu* (acyclic terpene utilization) gene cluster in P. aeruginosa and *P. citronellolis* (23). We note that in general, DSF is structurally more similar to conventional fatty acid molecules than acyclic terpenes. Interestingly, RdmA may not be the only transcriptional regulator associated with DSF metabolism. Transcriptome analysis revealed that there are about 20 regulatory genes showing significant changes at the transcript level in the presence of DSF, and deletion analysis identified 8 regulatory genes which could modulate the expression of the *dig* genes. These findings suggested that different bacterial species may adopt different molecular mechanisms in regulation of fatty acid metabolism, and strain HS-18 may have evolved complicated regulatory networks in modulation of DSF degradation.

Characterization of *rdmA* in strain HS-18 led to identification of the *dmg* cluster, which is located adjacent to *rdmA* and shares a high similarity to the *atu* cluster. Genetic analysis validated the key roles of the *dmg* genes in DSF degradation. The *atu* cluster was originally reported to be responsible for acyclic terpene utilization in P. aeruginosa and *P. citronellolis* (22, 23, 29). In the *atu* cluster, *atuA* was speculated to encode a 3-hydroxy-3-iso-hexenyl-glutaryl-CoA acet-atelyase (23). *atuB*, *atuD*, and *atuG* encode putative dehydrogenases or desaturases (30). *atuC* and *atuF* encode the two subunits of geranyl-CoA carboxylase. AtuE functions as an isohexenylglutaconyl-CoA hydratase and is responsible for the hydration of isohexenyl-glutaconyl-CoA to 3-hydroxy-3-isohex-enyl- glutaryl-CoA (24). AtuH has been suggested to be important to activate citronellate (a kind of acyclic terpene) to the corresponding thioester. Given the similarities in the peptide sequences, we believe that the previously characterized four fatty acyl-CoA ligases DigA–D, which are FadD homologues, are most likely responsible for activation of DSF to generate the DSF-CoA derivative, which is then further catabolized by DmgABCDEFGH (Fig. S3B). This is reminiscent of P. aeruginosa PAO1, in which multiple *fadD* genes and the *atu* cluster have been shown to be involved in acyclic terpene and fatty acid utilization (31). Interestingly, deletion of the *dig* genes resulted in a more greater impact in compromising DSF degradation capacity of strain HS-18 than deletion of the *dmg* genes (Fig. 4C). These findings suggest possible involvement of other enzymes in DSF metabolism, which deserves further investigation.

10.1128/mbio.03010-22.7 FIG S3 Proposed regulatory pathway mediated by RdmA and putative catabolic pathway of DSF degraded by Dmg proteins in strain HS-18. (A) Proposed regulatory pathway of *digA*–*D* and *dmgA*–*H* mediated by RdmA. (B) Proposed roles of the *dmg* cluster products from strain HS-18 in DSF degradation. An explicit step and unclear step are indicated by solid and dotted lines, respectively. Download FIG S3, TIF file, 1.8 MB. Copyright © 2022 Wang et al. 2022 Wang et al. https://creativecommons.org/licenses/by/4.0/ This content is distributed under the terms of the Creative Commons Attribution 4.0 International license.

The results from this study showed that RdmA acts as a repressor in modulating the transcriptional expression of the *dmg* and *dig* genes. DSF effectively inhibited RdmA binding to the *dmg* promoter and dissociate the RdmA-promoter complex in a concentration-dependent pattern (Fig. 6). This indicated that DSF is the effective signal ligand interacting with and modulating the functionality of RdmA. DSF family signals are fatty acid derivatives that differ in chain length and methyl substitution but contain a conserved unsaturated bond in the C-2 to C-3 position (4). Interestingly, compared with other fatty acid derivatives, the DSF family signals tested in this study, including DSF, PDSF, and BDSF, were much more active in induction of the transcriptional expression of the *dig* and *dmg genes* (Fig. 7B). These results suggested that RdmA can interact with a range of fatty acid molecules but prefers the DSF family signals DSF, BDSF, and PDSF as its ligands in modulation of the transcriptional expression of the *dig* and *dmg* genes.

The homologues of *rdmA* and the *dmg* cluster and the same gene arrangement were discovered not only in the genome of a wide range of Pseudomonas strains, including Pseudomonas nitroreducens, Pseudomonas denitrificans, Pseudomonas fluorescens, Pseudomonas nicosulfuronedens, Pseudomonas multiresinivorans, Pseudomonas taiwanensis, and Pseudomonas thermotolerans, but also in some other microorganisms, such as Acinetobacter, Enterobacter, *Hahella*, *Mangrovitalea*, and the Gram-positive bacterium Streptococcus, with sequence similarities ranging from 56.33% to 99.63% (Fig. S4). The palindromic motifs (AGCAAGCGCTTGG and CCAAGCGCTTGCT) which are vital for RdmA binding to the promoter of *dmgA* were also found in the promoter regions of the *dmgA* homologues in various Pseudomonas species and the other bacterial species mentioned above (Fig. S5). In addition, the homologues of *digA–D* were also found in many Pseudomonas species and other microorganisms listed in Fig. S5, with protein sequence similarities from 22% to 98% at the amino acid level. These findings suggest that RdmA mediating the Dig-Dmg DSF degradation pathway might be common in various microorganisms.

10.1128/mbio.03010-22.8 FIG S4 Conservation of the *dmg* cluster in other bacterial species. (A) Color presentation of *rdmA* and *dmg* genes. (B) Genetic organization of the *dmg* cluster in *P. nitroreducens* HS-18 with its homologues in other bacterial species. The predicted gene products of the *dmg* cluster from strain HS-18 are listed in Table S3, and their putative roles in DSF degradation are illustrated here. Download FIG S4, TIF file, 1.8 MB. Copyright © 2022 Wang et al. 2022 Wang et al. https://creativecommons.org/licenses/by/4.0/ This content is distributed under the terms of the Creative Commons Attribution 4.0 International license.

**FIG 8:**
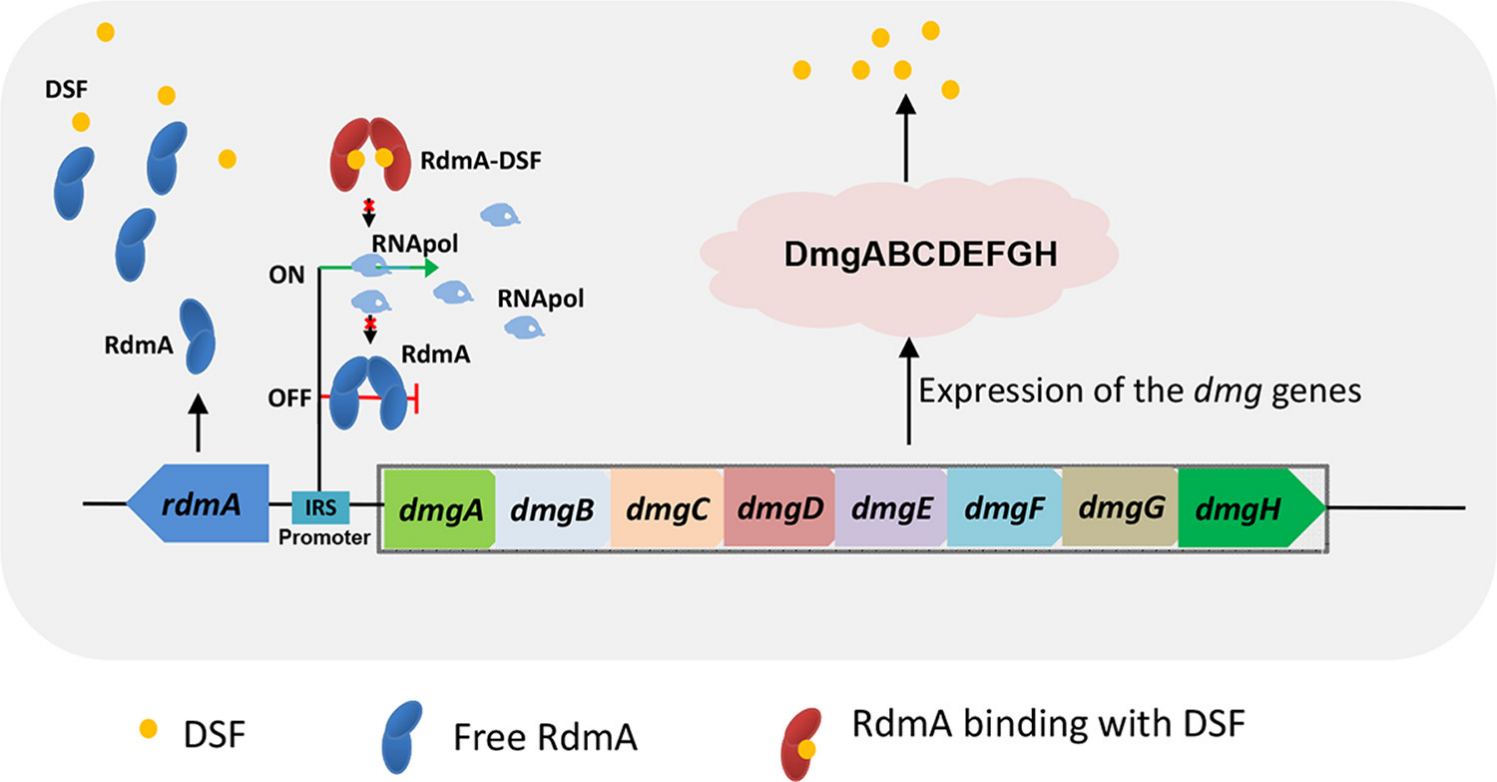
Proposed regulatory model of the *dmg* cluster via the quorum-sensing signal DSF positive feedback mechanism. In the absence of DSF, RdmA dimer binds to the inverted repeat sequences in the promoter and represses transcriptional expression of the *dmg* genes. In the presence of DSF, the *dmg* genes are expressed, as the negative regulator RdmA can not bind to the *dmg* promoter, likely due to formation of a RdmA-DSF complex.

In summary, the results from this study outline regulatory and metabolic pathways of DSF degradation in *P. nitroreducens* HS-18 (Fig. 8). In the absence of DSF, the negative regulator RdmA recognized and bound to the two repeated sequences of the *dmg* promoter and likely the promoter of unknown regulatory genes that control *dig* expression, blocking RNA polymerase interaction with the promoter and consequently transcription of the *dmg* and *dig* genes. In the presence of DSF, the binding of DSF to RdmA caused protein conformational changes and dissociation of the RdmA-promoter complex, allowing RNA polymerase to initiate the transcription of the *dmg* and *dig* genes. These original findings depict a novel and likely widely conserved regulator and mechanism with which microorganisms can modulate their QQ activity and provide useful clues and a genetic basis for further improving the bacterial QQ potency against the widely conserved DSF family of QS signals.

## MATERIALS AND METHODS

### Bacterial strains, plasmids, and culture conditions.

The bacterial strains and plasmids used in this study are listed in Table S1A in the supplemental material. *P. nitroreducens* HS-18 and its derivatives were grown at 30°C in Luria-Bertani (LB) medium (10 g/liter tryptone, 5 g/liter yeast extract, 10 g/liter NaCl; pH 7.0) or on Pseudomonas isolation agar (Difco) plates, unless otherwise indicated. E. coli strains were grown at 37°C in LB medium. For β-galactosidase assays, RNA isolations, and DSF degradation assays, a modified minimal medium (MM*) was used (15). When necessary, antibiotics were added at a final concentration of 50 μg/mL for gentamicin and kanamycin and 100 μg/mL for ampicillin. Bacterial growth was determined by measuring the optical density at a wavelength of 600 nm (OD_600_). Synthetic DSF (*cis*-11-methyl-2-dodecenoic acid) was dissolved in methanol to prepare a stock solution (100 mM), which was kept refrigerated for subsequent experiments.

### Construction of in-frame deletion mutants, complementation, and overexpression strains.

Wild-type strain *P. nitroreducens* HS-18 and the reporter strain HS-18 (*digA*::*lacZ*), which was generated in our previous study (15), were used as the parental strains for generation of deletion mutants. All in-frame deletion (markerless) mutants of the candidate genes were constructed using the suicide vector pK18mob*sacB* via a homologous double-crossover method as described previously (15).

For complementation and overexpression, the gene coding region and the predicted ribosomal binding site was amplified by PCR with the primers listed in Table S1B. The PCR fragments were cloned into the vector pBBR1-MCS2 to generate corresponding expression constructs, which were then transformed into the deletion mutants or wild-type strain by triparental mating with the helper strain E. coli HB101(pRK2013). Transformants were selected on LB agar containing 50 μg/mL kanamycin and verified by PCR and DNA sequencing.

### β-Galactosidase assay.

Expression of the *digA* gene, which is inducible by DSF, was determined by measurement of the β-galactosidase activities of strain HS-18 (*digA*::*lacZ*) and its derivatives, as described previously (15). Briefly, the overnight cultures in LB were resuspended with fresh MM* to an OD_600_ of 1.0 and then inoculated at a 1:100 ratio into 0.5 mL of fresh MM* supplemented with DSF at a final concentration of 0.5 mM. The β-galactosidase activity was measured using a chemiluminescent microtiter dish assay (Tropix Galacto-LightPlus; Applied Biosystems) after incubation at 30°C and 200 rpm for 6 h. The assays were carried out in triplicate and repeated at least three times.

### DSF degradation assay.

Detection of DSF degradation activity was performed using MM* plates containing 5 mM DSF as described previously (15). Briefly, a 0.5-μL aliquot of fresh bacterial culture at an OD_600_ of ≈0.5 was spotted onto the plates, and the clear zones produced by the bacterial colonies were observed after incubation for 7 days. For quantitative measurement of DSF in liquid culture, overnight cultures adjusted to the same OD_600_ of ≈0.5 were inoculated at a 1:100 ratio into the MM* medium supplemented with DSF at a final concentration of 0.5 mM. The MM* medium supplemented with 0.5 mM DSF was used as a reference control. The culture supernatants were then collected after incubation for 9 h and extracted with ethyl acetate. The organic solvent was evaporated and the residues were dissolved in 200 μL methanol. A 30-μL aliquot of the extracted sample was applied to an HPLC C_18_ reverse-phase column and eluted with methanol and water (80:20 [vol/vol] water containing 0.1% formic acid) at a flow rate of 1 mL/min. The relative amount of the remaining DSF was estimated by comparing the peak area of treatment to that for the reference control. The peak area was monitored using a diode array detector (Agilent 1200 Infinity series) at a UV wavelength of 210 nm by using pure DSF as the reference control.

### RNA preparation, RNA-Seq, and RT-qPCR analysis.

For RNA sequencing (RNA-Seq) analysis, overnight culture of wild-type strain HS-18 were diluted to an OD_600_ of 0.5 with liquid MM* medium and then inoculated at a 1:100 ratio into the same medium containing 10 mM succinate with or without 0.5 mM DSF. The cultures were incubated at 30°C with shaking at 150 rpm for 5 h; samples were then taken and pelleted to harvest the bacterial cells. Total RNAs were isolated using the Ribopure RNA extraction kit (Ambion, Life Technologies) according to the manufacturer’s protocol. Ribosomal RNAs were removed with a Ribo-zero rRNA removal kit (Gram-negative bacteria; Illumina, Madison, WI, USA). cDNA library preparation and RNA sequencing were performed by Novogene (Beijing, China) using an Illumina HiSeq 2500/MiSeq system. Clean reads were obtained by removing adaptors, unknown nucleotides, and low-quality reads prior to alignment with the *P. nitroreducens* HS-18 genome (GenBank accession numbers CP084413.1 and CP084414.1), using Bowtie2 v. 2.2.3 (32). The gene expression level was normalized using the method of fragments per kilobase of transcript per million mapped reads (FPKM) (33). DESeq2 was used for differential expression analysis (34), using the Benjamini-Hochberg adjustment for multiple comparisons and a false-discovery rate of <0.05. The differentially expressed genes were then subjected to enrichment analysis through the Gene Ontology and Kyoto Encyclopedia of Genes and Genomes pathway databases.

For RT-qPCR analysis, RNA preparation of wild-type strain HS-18 and its mutant Δ*rdmA* in liquid MM* with or without 0.5 mM DSF followed the method described above. The residue DNA was removed by treatment with DNase for 40 min at 37°C. Three biological replicates were used for each sample. The cDNA samples were generated with 400 ng total RNA sample as template by using the EasyScript one-step genomic DNA removal and cDNA synthesis SuperMix kit (TransGen, Beijing, China) according to the manufacturer’s protocol. RT-qPCR analysis of target genes was performed using a Quant Studio 6 Flex system with the primer pairs listed in Table S1B and the talent qPCR PreMix (SYBRgreen) kit (Tiangen Biotech) following the manufacturer’s protocol. The constitutively expressed *rpoD* gene was used as a reference to standardize all samples and replicates.

### Protein purification.

The N-terminal His_6_-tagged recombinant RdmA protein was prepared by construction of the recombinant expression vector *rdmA*-pET32a with the primers listed in Table S1B and was transformed into E. coli BL21(DE3). Isopropyl-β-d-thiogalactopyranoside was added at a final concentration of 0.5 mM to induce protein expression when the optical density (OD_600_) of the bacterial culture reached ~0.6. The culture was further incubated for 16 h at 18°C with shaking at 200 rpm. The His_6_-tagged proteins were then purified using Ni^2+^-nitrilotriacetic acid affinity resin following the manufacturer’s protocol (TransGen Biotech). Purified proteins were resuspended in phosphate-buffered saline for subsequent analysis.

### Electrophoretic mobility shift assay.

All the DNA fragments used in the EMSA were amplified with the genomic DNA of strain HS-18 as template using the primers listed in Table S1B and were then purified and labeled with a biotin 3′-end DNA labeling kit (Thermo). EMSA was performed using the LightShift chemiluminescent EMSA kit (Thermo Scientific Pierce) in a 10-μL reaction mixture containing 2 nM of labeled DNA fragment (20 fmol) and varied amounts of purified RdmA (0 to 4 μM), following the manufacturer’s protocol. After incubation at 22°C for 30 min, the reaction products were separated with a 0.5× Tris-borate-EDTA buffer on 6.4% nondenaturing polyacrylamide and electrophoresed at 100 V (constant voltage) for 2 h in an ice bath, prior to chemiluminescence detection. Labeled DNA fragments incubated without RdmA protein were included as a negative control.

DSF, several fatty acid structural analogues (PDSF, BDSF, lauric acid, myristic acid, palmitic acid, linoleic acid, oleic acid, and erucic acid), and hexadecane were tested as potential ligands by EMSA. Two different treatments were used to validate the role of DSF. The first method involved incubation of RdmA with DSF at 30°C for 30 min prior to P*_dmgA_* addition and 30 min of incubation at 22°C. The second method involved coincubation of RdmA and P*_dmgA_* at 22°C for 30 min prior to addition of DSF and incubation at 30°C for 30 min. The effects of other potential ligands on binding of RdmA and P*_dmgA_* were assayed using the second method. The solvent methanol solution was included as a negative control.

### Data availability.

The raw data from our transcriptome analyses were deposited in the NCBI Sequence Read Archive under accession number PRJNA883994.
