# Supplementary material for: RdmA Is a Key Regulator in Autoinduction of DSF Quorum Quenching in Pseudomonas nitroreducens HS-18
Source: mBio. 2022 Dec 20;14(1):e03010-22. doi: 10.1128/mbio.03010-22 (PMC9973270; doi:10.1128/mbio.03010-22)
Supplement: TABLE S2 [file mbio.03010-22-s0007.docx]

**Table S2** Differentially expressed regulatory genes in the presence and absence of DSF

| Gene | Log_2_ fold change | Annotation |
| --- | --- | --- |
| HS.18_GM000244 | 1.4474 | Transcriptional regulatory protein FlbD |
| HS.18_GM000782 | 2.6855 | HTH-type transcriptional repressor KstR2 |
| HS.18_GM001642 | 3.3776 | Uncharacterized HTH-type transcriptional regulator YdeS |
| HS.18_GM001988 | 2.1263 | Virulence transcriptional regulatory protein PhoP |
| HS.18_GM002215 | 1.772 | HTH-type transcriptional regulator PetP |
| HS.18_GM002462 | 1.6278 | Putative sigma L-dependent transcriptional regulator YqiR |
| HS.18_GM002468 | 4.2928 | HTH-type transcriptional regulator CueR |
| HS.18_GM002469 | 3.8836 | Signal-transduction and transcriptional-control protein |
| HS.18_GM002507 | 2.1624 | Ornithine utilization regulator |
| HS.18_GM003304 | 1.0042 | Uncharacterized HTH-type transcriptional regulator YbhD |
| HS.18_GM003305 | 1.1376 | Transcriptional repressor IclR |
| HS.18_GM003698 | 1.2815 | Arginine utilization regulatory protein RocR |
| HS.18_GM003900 | 1.4719 | Transcriptional activator protein CopR |
| HS.18_GM003981 | 2.6881 | HTH-type transcriptional repressor KstR2 |
| HS.18_GM003994 | 2.9845 | Transcriptional regulatory protein RcsB |
| HS.18_GM004051 | 1.4602 | Probable HTH-type transcriptional regulator YttP |
| HS.18_GM001262 | -1.5748 | Transcriptional regulatory protein RcsB |
| HS.18_GM001932 | -1.1818 | Transcriptional regulatory protein OmpR |
| HS.18_GM003283 | -4.4881 | Transcriptional activator protein ExaE |
| HS.18_GM003877 | -2.984 | Virulence factors putative positive transcription regulator BvgA |
