# Supplementary figures and images for: RdmA Is a Key Regulator in Autoinduction of DSF Quorum Quenching in Pseudomonas nitroreducens HS-18
Source: mBio. 2022 Dec 20;14(1):e03010-22. doi: 10.1128/mbio.03010-22 (PMC9973270; doi:10.1128/mbio.03010-22)

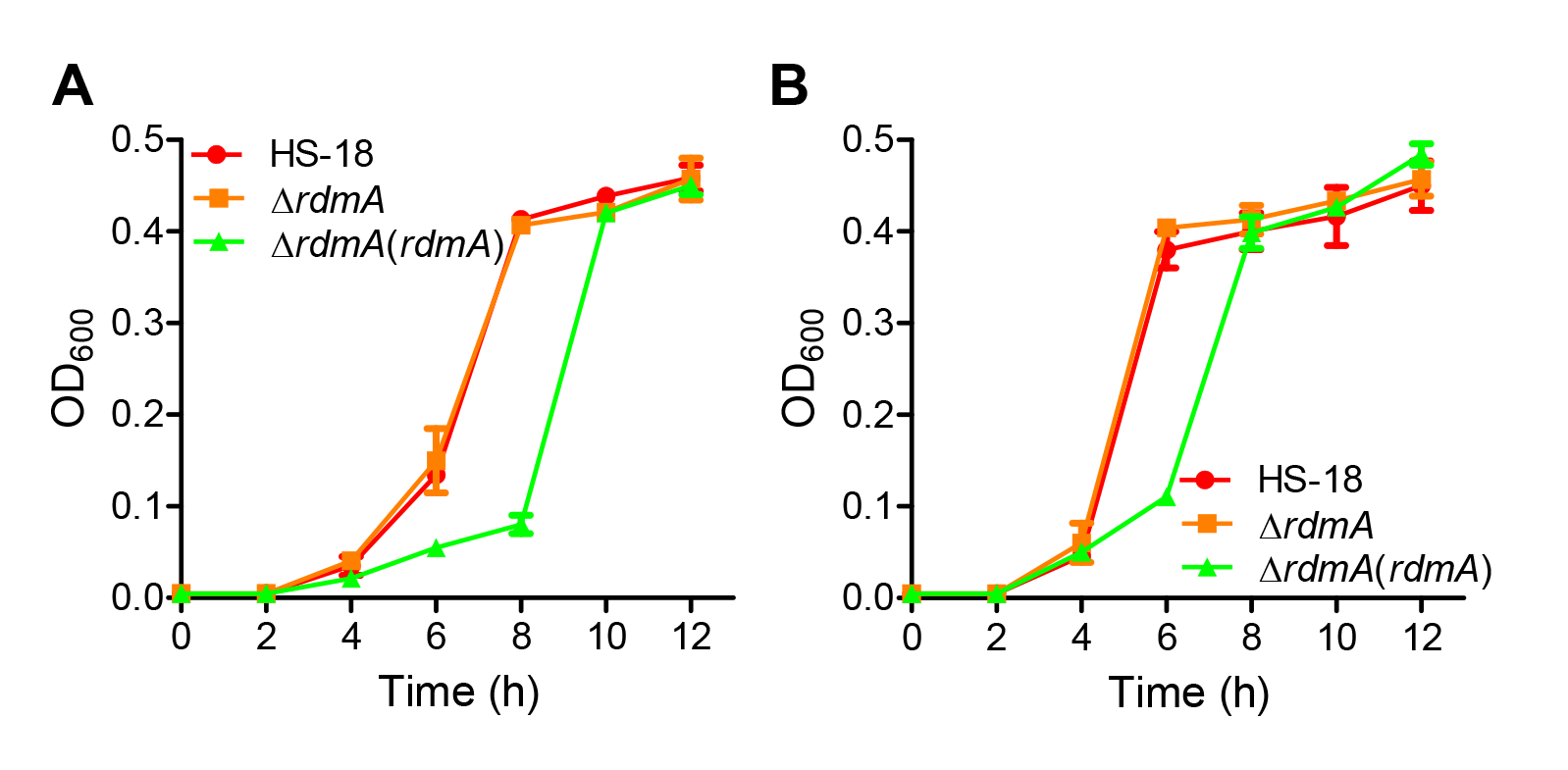

Supplement: FIG S1 [file mbio.03010-22-s0001.tif]

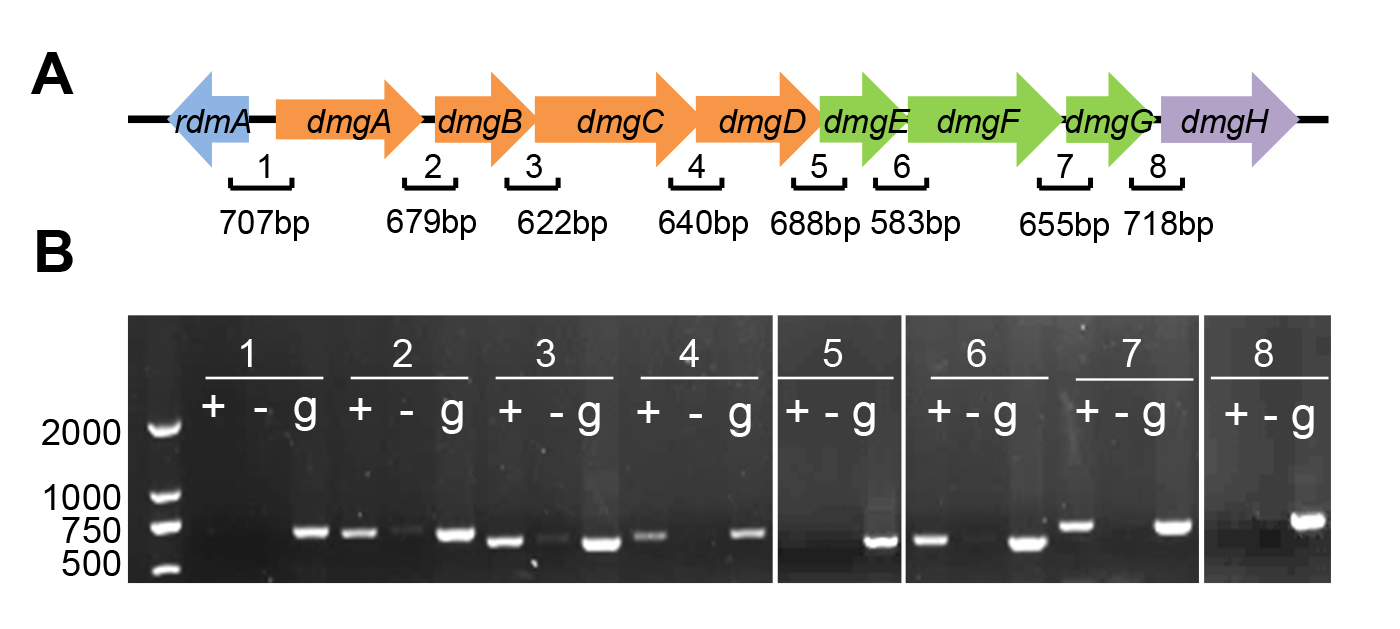

Supplement: FIG S2 [file mbio.03010-22-s0002.tif]

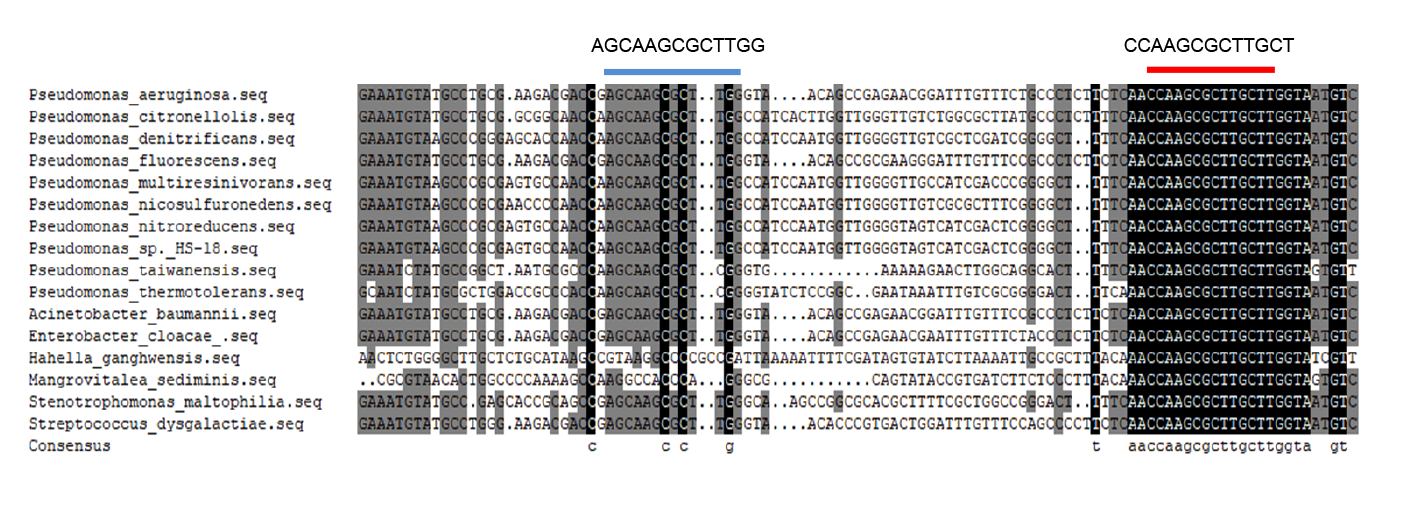

Supplement: FIG S5 [file mbio.03010-22-s0005.tif]

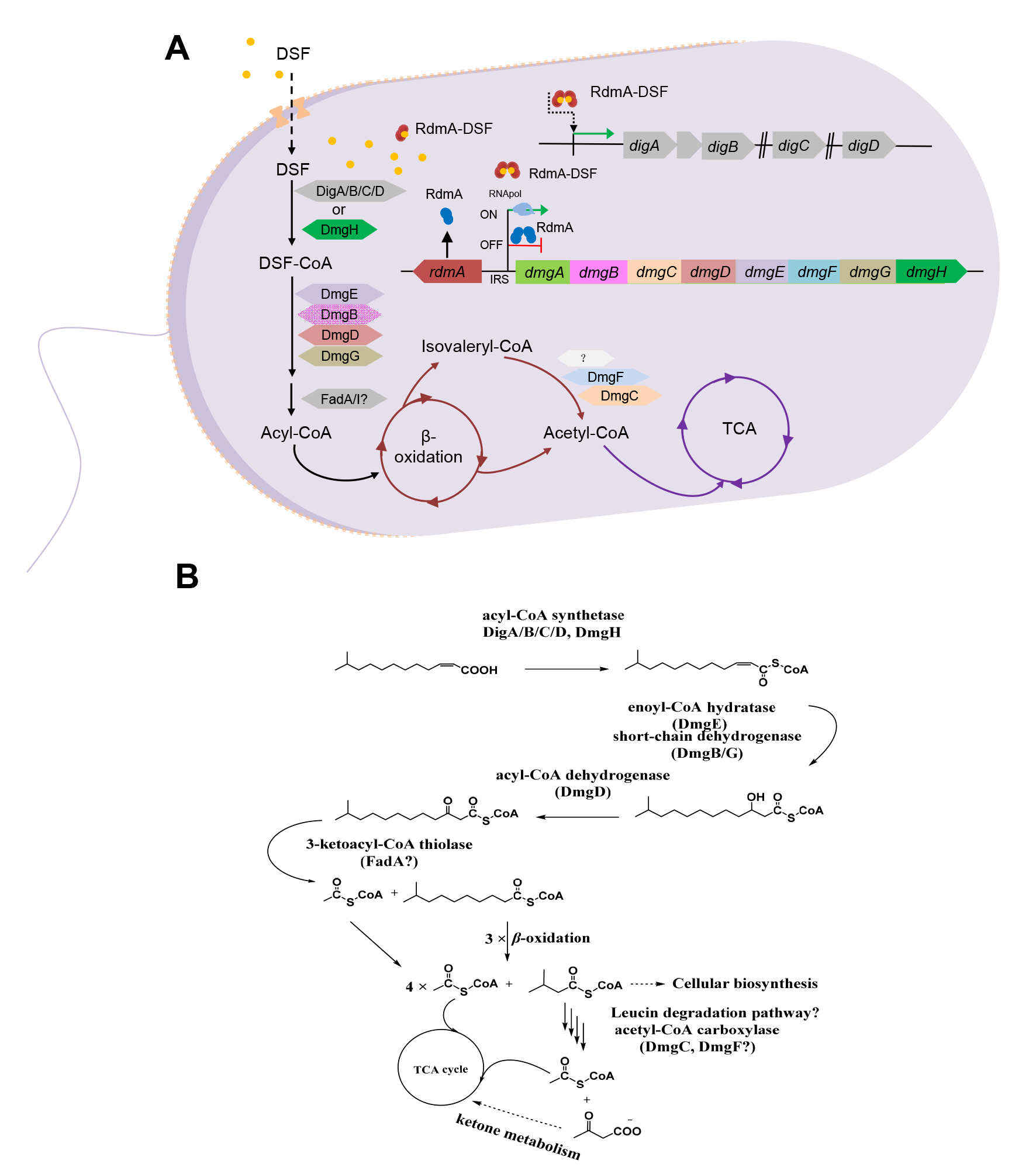

Supplement: FIG S3 [file mbio.03010-22-s0003.tif]

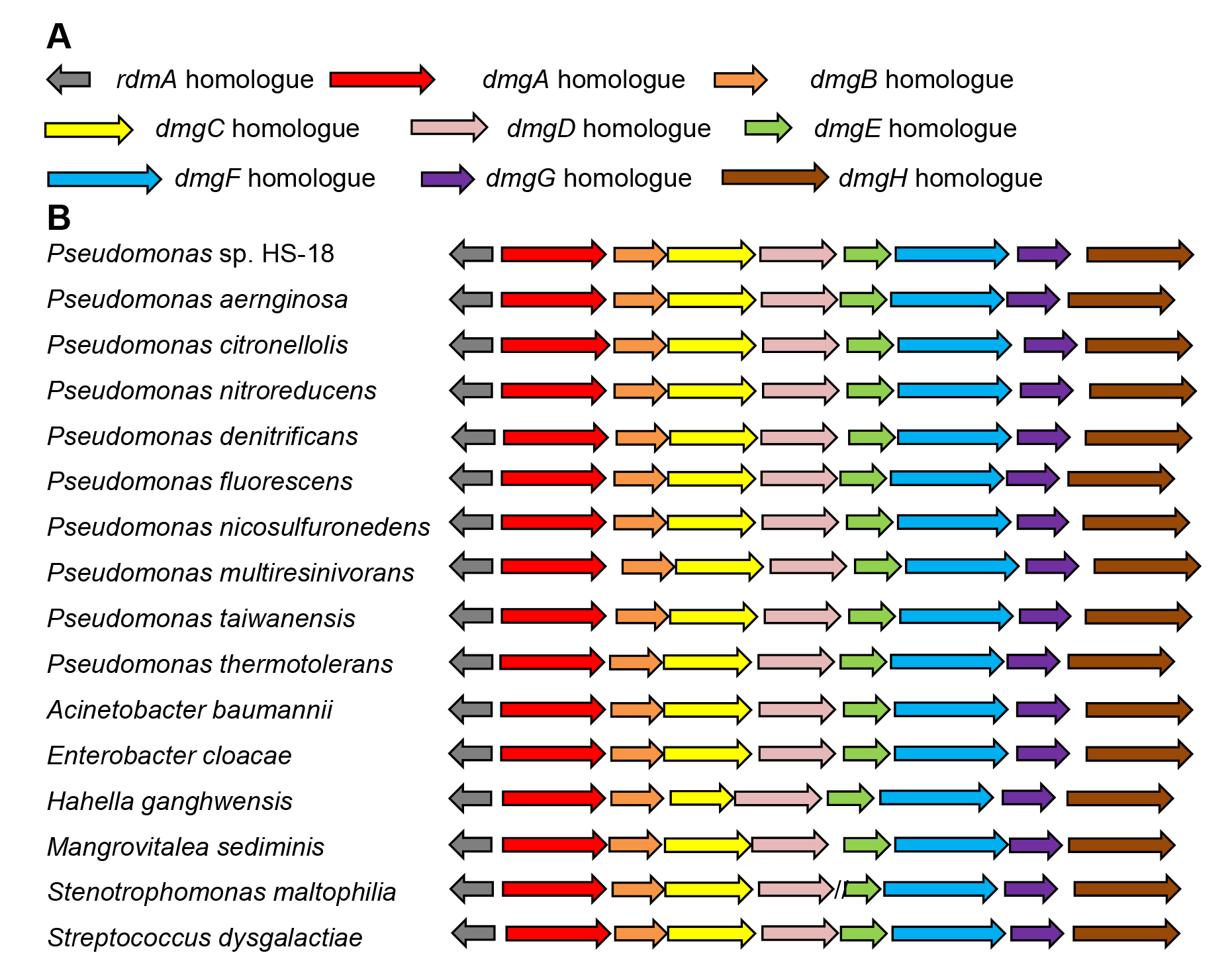

Supplement: FIG S4 [file mbio.03010-22-s0004.tif]
