# Supplementary material for: RdmA Is a Key Regulator in Autoinduction of DSF Quorum Quenching in Pseudomonas nitroreducens HS-18
Source: mBio. 2022 Dec 20;14(1):e03010-22. doi: 10.1128/mbio.03010-22 (PMC9973270; doi:10.1128/mbio.03010-22)
Supplement: TABLE S3 [file mbio.03010-22-s0008.docx]

**Table S3** The similarities of the proteins encoded by the *dmg* cluster in strain HS-18 to the corresponding counterparts in *P*. *aeruginosa* PAO1

| *P*. sp. HS-18 | | *P*. *aeruginosa* PAO1 | | Identities  (%) | The *rdmA* and *dmg* genes Log_2_ fold changes  in RNAseq results | Putative functions |
| --- | --- | --- | --- | --- | --- | --- |
| Name/MW(kDa) | | Name/MW(kDa) | |  |  |  |
| RdmA | 23.21 | AtuR | 22.06 | 86.29 | 2.6881 | Probable terR family transcription regulator |
| DmgA | 63.26 | AtuA | 64.46 | 83.92 | 5.1746 | Hypothetical protein |
| DmgB | 60.56 | AtuB | 30.75 | 90.38 | 4.158 | Probable short-chain dehydrogenase |
| DmgC | 57.17 | AtuC | 57.34 | 91.08 | 3.9163 | Probable acetyl-CoA carboxylase |
| DmgD | 42.76 | AtuD | 42.72 | 87.82 | 3.029 | Probable acyl-CoA dehydrogenase |
| DmgE | 27.52 | AtuE | 27.72 | 81.68 | 2.842 | Probable enoyl-CoA hydratase |
| DmgF | 71.33 | AtuF | 71.76 | 79.06 | 2.5833 | Probable acetyl/propionyl-CoA carboxylase |
| DmgG | 31.17 | AtuG | 29.63 | 87.23 | 2.0831 | Probable short-chain dehydrogenase |
| DmgH | 66.82 | AtuH | 67.66 | 82.24 | - | Probable fatty acid-CoA ligase |
