# Supplementary material for: RdmA Is a Key Regulator in Autoinduction of DSF Quorum Quenching in Pseudomonas nitroreducens HS-18
Source: mBio. 2022 Dec 20;14(1):e03010-22. doi: 10.1128/mbio.03010-22 (PMC9973270; doi:10.1128/mbio.03010-22)
Supplement: TABLE S1 [file mbio.03010-22-s0006.docx]

**Table S1A** Bacterial strains used in this study

| Strain or plasmid | Description | Source or reference |
| --- | --- | --- |
| Strains |  |  |
| *Escherichia coli* |  |  |
| E. coli HB101 (pRK2013) | Kan^R^, helper strain in triparental mating | Lab collection |
| DH5α | F^-^ *deoR* *endA1 gyrA96 hsdR17*(r_K_^-^m_K_^+^) *recA1* *relA1* *supE*44 *thi-1* Δ(*lacZYA-argF*)*U*169(φ80*lacZ*ΔM15) | TransGen Biotech |
| DH5α (pBBR1-2) | DH5α harboring the plasmid pBBR1-2*,* Kan^R^ | This study |
| DH5α (*rdmA*) | DH5α harboring the *digA* expression plasmid pBBR1-*rdmA,* Km^R^ | This study |
| DH5α (pk18-*rdmA*) | DH5α harboring the pK18 plasmid ligated with the up arm and down arm of *rdmA*, Gen^R^ | This study |
| BL21 (DE3) | F^-^ *dcm* *omp T* *hsdS*(r_B_^-^m_B_^-^) *gal* (λDE3) | TransGen Biotech |
| BL21 (DE3) (*rdmA*) | BL21(DE3) harboring the *rdmA* expression plasmid pET32a-*rdmA*, Amp^R^ | This study |
| *Pseudomonas* sp. HS-18 |  |  |
| HS-18 | Wild type, Tc^R^ | This study |
| HS-18 (*digA*::*lacZ*) | The knock-in reporter in HS-18 by placing *lacZ* in the genome under the control of *digA* promoter, Tc^R^ | This study |
| ΔHS.18_GM000244 | The gene HS.18_GM000244 in-frame deletion mutant in HS-18 (*digA*::*lacZ*), Tc^R^ | This study |
| ΔHS.18_GM000612 | The gene HS.18_GM000612 in-frame deletion mutant in HS-18 (*digA*::*lacZ*), Tc^R^ | This study |
| ΔHS.18_GM000728 | The gene HS.18_GM000728 in-frame deletion mutant in HS-18 (*digA*::*lacZ*), Tc^R^ | This study |
| ΔHS.18_GM001642 | The gene HS.18_GM001642 in-frame deletion mutant in HS-18 (*digA*::*lacZ*), Tc^R^ | This study |
| ΔHS.18_GM001932 | The gene HS.18_GM001932 in-frame deletion mutant in HS-18 (*digA*::*lacZ*), Tc^R^ | This study |
| ΔHS.18_GM001988 | The gene HS.18_GM001988 in-frame deletion mutant in HS-18 (*digA*::*lacZ*), Tc^R^ | This study |
| ΔHS.18_GM002215 | The gene HS.18_GM002215 in-frame deletion mutant in HS-18 (*digA*::*lacZ*), Tc^R^ | This study |
| ΔHS.18_GM002462 | The gene HS.18_GM0002462 in-frame deletion mutant in HS-18 (*digA*::*lacZ*), Tc^R^ | This study |
| ΔHS.18_GM002468 | The gene HS.18_GM002468 in-frame deletion mutant in HS-18 (*digA*::*lacZ*), Tc^R^ | This study |
| ΔHS.18_GM002469 | The gene HS.18_GM002469 in-frame deletion mutant in HS-18 (*digA*::*lacZ*), Tc^R^ | This study |
| ΔHS.18_GM002507 | The gene HS.18_GM002507 in-frame deletion mutant in HS-18 (*digA*::*lacZ*), Tc^R^ | This study |
| ΔHS.18_GM003283 | The gene HS.18_GM003283 in-frame deletion mutant in HS-18 (*digA*::*lacZ*), Tc^R^ | This study |
| ΔHS.18_GM003304 | The gene HS.18_GM003304 in-frame deletion mutant in HS-18 (*digA*::*lacZ*), Tc^R^ | This study |
| ΔHS.18_GM003305 | The gene HS.18_GM000244 in-frame deletion mutant in HS-18 (*digA*::*lacZ*), Tc^R^ | This study |
| ΔHS.18_GM003698 | The gene HS.18_GM003698 in-frame deletion mutant in HS-18 (*digA*::*lacZ*), Tc^R^ | This study |
| ΔHS.18_GM003877 | The gene HS.18_GM003877 in-frame deletion mutant in HS-18 (*digA*::*lacZ*), Tc^R^ | This study |
| ΔHS.18_GM003900 | The gene HS.18_GM003900 in-frame deletion mutant in HS-18 (*digA*::*lacZ*), Tc^R^ | This study |
| ΔHS.18_GM003981 | The gene HS.18_GM003981 in-frame deletion mutant in HS-18 (*digA*::*lacZ*), Tc^R^ | This study |
| ΔHS.18_GM003994 | The gene HS.18_GM003994 in-frame deletion mutant in HS-18 (*digA*::*lacZ*), Tc^R^ | This study |
| ΔHS.18_GM004501 | The gene HS.18_GM004501 in-frame deletion mutant in HS-18 (*digA*::*lacZ*), Tc^R^ | This study |
| Δ*rdmA* | The *rdmA* in-frame deletion mutant in HS-18, Tc^R^ | This study |
| Δ*rdmA* (*rdmA*) | The complementation of *rdmA* in deletion mutant △*rdmA* harboring plasmid pBBR1- *rdmA*, Tc^R^; Km^R^ | This study |
| WT (*rdmA*) | The overexpression of *rdmA* in wild strain HS-18 harboring plasmid pBBR1-*rdmA*, Tc^R^; Km^R^ | This study |
| Plasmids |  |  |
| pBBR1-MCS2 | Km^R^, road-host-range cloning vector | Lab collection |
| pK18mob*scaB* | Gen^R^, *sacB*-based gene replacement vector | Lab collection |
| pET32a | Amp^R^,T7 promoter-based expression vector | Lab collection |

**Table S1B** Primers used in this study

| Primer name | Primer sequence (5′to 3′) | | Digestion sites ^a^ |
| --- | --- | --- | --- |
| pk18-F | TGCTTCCGGCTCGTATGTTG | |  |
| pk18-R | GCGAAAGGGGGATGTGCTG | |  |
| MCS-F | GGCTCGTATGTTGTGTGG | |  |
| MCS-R | TCTTCGCTATTACGCCAGCT | |  |
| R0244-KO-up-F | gagctcggtacccggggatccAGATCTCTTTGATGTACTGCGGAA | | *BamH*Ⅰ |
| R0244-KO-up-R | aatcagaagtgcacCAGCGTCTGCAAAAGGAGGG | |  |
| R0244-KO-up-F | acgctgGTGCACTTCTGATTTCGTCTTCC | |  |
| R0244-KO-up-R | acgacggccagtgccaagcttCTTCACGCCCTTGGCCGC | | *Hind*III |
| R0612-KO-up-F | gagctcggtacccggggatccGGCCGACAGTTCGACCGC | | *BamH*Ⅰ |
| R0612-KO-up-R | gtgccGGTAATAGCTACCATGCCCGAG | |  |
| R0612-KO-up-F | atggtagctattaccGGCACCCGCTGAAGCATC | |  |
| R0612-KO-up-R | acgacggccagtgccaagcttAGAAGGCTTCCCCGAGCA | | *Hind*III |
| R0782-KO-up-F | gagctcggtacccggggatccGCGACCGCTTCAACCTCG | | *BamH*Ⅰ |
| R0782-KO-up-R | atcacaggcgtggGGTTTGCCGGTAGGCCAT | |  |
| R0782-KO-dn-F | gcaaaccCCACGCCTGTGATGGAGGA | |  |
| R0782-KO-dn-R | acgacggccagtgccaagcttGAACTCGTTGCGGCTGCC | | *Hind*III |
| R1642-KO-up-F | gagctcggtacccggggatccCGACGCTCACCGCCTCAC | | *BamH*Ⅰ |
| R1642-KO-up-R | cGTTCGATGGCGACATGATTTT | |  |
| R1642-KO-dn-F | aatcatgtcgccatcgaacGCCTTCAGGCCCTGACTCG | |  |
| R1642-KO-dn-R | acgacggccagtgccaagcttCGGCTCACCGCCGCCAGT | | *Hind*III |
| R1932KO-up-F | gagctcggtacccggggatccGTGAACGACTTCAGCACCATGG | | *BamH*Ⅰ |
| R1932-KO-up-R | tcatggccGGCAGGCTGGTTCACTTCG | |  |
| R1932-KO-up-F | aaccagcctgccGGCCATGAGTGAGCGCCG | |  |
| R1932-KO-up-R | acgacggccagtgccaagcttGCTGAACAGCTGGCCACG | | *Hind*III |
| R1988-KO-up-F | gagctcggtacccggggatccAAAATCGCCCTCGCCAGC | | *BamH*Ⅰ |
| R1988-KO-up-R | tacacagctcCAGCAGTTTCATGCGCGTG | |  |
| R1988-KO-dn-F | gaaactgctgGAGCTGTGTAAATGATCCGCTCG | |  |
| R1988-KO-dn-R | acgacggccagtgccaagcttGAATTGCGATAGCGCTCGC | | *Hind*III |
| R2215-KO-up-F | gagctcggtacccggggatccCCGGCAGCAGCGTCTTGG | | *BamH*Ⅰ |
| R2215KO-up-R | ggGTCAGGCATATTGACGTATCCATC | |  |
| R2215-KO-dn-F | tacgtcaatatgcctgacCCGGACGATTAGCGGACG | |  |
| R2215-KO-dn-R | acgacggccagtgccaagcttCAGTATGTTGCACACTCCCGAC | | *Hind*III |
| R2462-KO-up-F | gagctcggtacccggggatccTCCACGACACCTATGGCCAG | | *BamH*Ⅰ |
| R2462-KO-up-R | tttcaggactgaagGGTGTCGGTCATGGTCAGGC | |  |
| R2462-KO-dn-F | gacaccCTTCAGTCCTGAAAAAGAGACAAGTC | |  |
| R2462-KO-dn-R | acgacggccagtgccaagcttCGCTGGCTCACCTGGCGC | | *Hind*III |
| R2468-KO-up-F | gagctcggtacccggggatccTGCGGCCCCTCGATGGGC | | *BamH*Ⅰ |
| R2468-KO-up-R | GGTCGTGGGCATGGGAAT | |  |
| R2468-KO-dn-F | agattcccatgcccacgaccGCCACGAGCTGATCTCGCC | |  |
| R2468-KO-dn-R | acgacggccagtgccaagcttGCACTTCCACATCATCGAAGAA | | *Hind*III |
| R2469-KO-up-F | gagctcggtacccggggatccCCTCGGCCGTGTCCAGTT | | *BamH*Ⅰ |
| R2469-KO-up-R | agaacatcagcccacGGCTTCGCGCAACAGGTT | |  |
| R2469-KO-dn-F | aagccGTGGGCTGATGTTCTTCACCC | |  |
| R2469-KO-dn-R | acgacggccagtgccaagcttCTGCTGGCCGGCGGACTG | | *Hind*III |
| R2507-KO-up-F | gagctcggtacccggggatccCATGACCGAGACCAGCCCG | | *BamH*Ⅰ |
| R2507-KO-up-R | tttcaggctcCTTCTCTACGCCCATGGCG | |  |
| R2507-KO-dn-F | cgtagagaagGAGCCTGAAATTTAAGGGCGC | |  |
| R2507-KO-dn-R | acgacggccagtgccaagcttAGGCACCGAAGCGGCCCT | | *Hind*III |
| R3283-KO-up-F | gagctcggtacccggggatccGACCATGCCCTGCCCGAG | | *BamH*Ⅰ |
| R3283-KO-up-R | gatggcCAGCAGGATCTTCACGCCC | |  |
| R3283-KO-dn-F | tgaagatcctgctgGCCATCGCCGGCTAGCGT | |  |
| R3283-KO-dn-R | acgacggccagtgccaagcttGTTTGCCACGGTGCGCGA | | *Hind*III |
| R3304-KO-up-F | gagctcggtacccggggatccTTCTTCCACTTCGACGACGG | | *BamH*Ⅰ |
| R3304-KO-up-R | ccgctcCTTTACGGTCATTTATAAATACGCCA | |  |
| R3304-KO-dn-F | aaatgaccgtaaagGAGCGGGAGTGATCGCGC | |  |
| R3304-KO-dn-R | acgacggccagtgccaagcttCTGGGCGAGTACCTGGACATG | | *Hind*III |
| R3305-KO-up-F | gagctcggtacccggggatccGGCTGTCGAGCAGTTCGACG | | *BamH*Ⅰ |
| R3305-KO-up-R | gatctttccGTCGTTCATGGTCTTCAACTTAAAGG | |  |
| R3305-KO-dn-F | ccatgaacgacGGAAAGATCTGAGATTCCGCAA | |  |
| R3305-KO-dn-R | acgacggccagtgccaagcttATGGTCCGCAACCGCCGT | | *Hind*III |
| R3698-KO-up-F | gagctcggtacccggggatccTCCACGGTACCAGGGGCC | | *BamH*Ⅰ |
| R3698-KO-up-R | GAGCCAGTTCAACTCCTCATTTCG | |  |
| R3698-KO-dn-F | atgaggagttgaactggctcCCAGGACACTCATAGAGCCGC | |  |
| R3698-KO-dn-R | acgacggccagtgccaagcttTCGCCGGATCCATACGCG | | *Hind*III |
| R3877-KO-up-F | gagctcggtacccggggatccGTTGGCTGCTTCTGCACGC | | *BamH*Ⅰ |
| R3877-KO-up-R | cGATTCTGCTCATGCCACTTCTCA | |  |
| R3877-KO-dn-F | aagtggcatgagcagaatcGCCTCGTCCTGATCACGATACA | |  |
| R3877-KO-dn-R | acgacggccagtgccaagcttGATCCTGGCCTTCCTCTTCG | | *Hind*III |
| R3900-KO-up-F | gagctcggtacccggggatccCGAAGCGCACGGTCTCGC | | *BamH*Ⅰ |
| R3900-KO-up-R | GAGGAGTTTCATCCGGGTATCC | |  |
| R3900-KO-dn-F | atacccggatgaaactcctcGAGCGCGAAGCATGAGCG | |  |
| R3900-KO-dn-R | acgacggccagtgccaagcttAGGTTGCCGTGCAGCGCT | | *Hind*III |
| R3981-KO-up-F | gagctcggtacccggggatccGCGCGGCGCTGACCACCG | | *BamH*Ⅰ |
| R3981-KO-up-R | atcgcgcatcacGCGGTCATCCACGCTTTCT | |  |
| R3981-KO-dn-F | atgaccgcGTGATGCGCGATTGAGGC | |  |
| R3981-KO-dn-R | acgacggccagtgccaagcttCTGATCGGCGCGTTCTATGT | | *Hind*III |
| R3994-KO-up-F | gagctcggtacccggggatccCGAGTCGCTGGCCCGTGC | | *BamH*Ⅰ |
| R3994-KO-up-R | ccgagCTTTTCCGATGACATCACATTCTC | |  |
| R3994-KO-dn-F | atgtcatcggaaaagCTCGGGGAGATCTGAGCGC | |  |
| R3994-KO-dn-R | acgacggccagtgccaagcttGGTCGGATCGACCACGACA | | *Hind*III |
| R4501-KO-up-F | gagctcggtacccggggatccTGCCTTCGCGCTTGAAGC | | *BamH*Ⅰ |
| R4501-KO-up-R | tcagactttggcgggGGTTTCCGACTGGGCCAT | |  |
| R4501-KO- dn -F | aaaccCCCGCCAAAGTCTGATTTGC | |  |
| R4501-KO- dn -R | acgacggccagtgccaagcttGGCGGCCATCAGCTCGCG | | *Hind*III |
| R0244-KO-detect-F | | GCGAAACCGAACTTGCCCAG |  |
| R0244-KO-detect-R | | TTGTCGAACAGCTCGTAGCCG |  |
| R0612-KO-detect-F | | CTCATCAAATGCGGCTCTGC |  |
| R0612-KO-detect-R | | GCTGGAGCGTGCCAAGCG |  |
| R0782-KO-detect-F | | GGCAACAAGCGCAACATCG |  |
| R0782-KO-detect-R | | ACCAGTTGCCCAGCTTGTTC |  |
| R1642-KO-detect-F | | AATCTTGCCGCTGAAGCGC |  |
| R1642-KO-detect-R | | CGGTGCGGGTGGACTTCC |  |
| R1932-KO-detect-F | | CCAGCCGCTCTCTTCTGTCG |  |
| R1932-KO-detect-R | | GCGCAAGCGGGTGATCGGC |  |
| R1988-KO-detect-F | | CTCTGACGGGAGAATCCCC |  |
| R1988-KO-detect-R | | GTTTCGCTGACGCCCTGC |  |
| R2215-KO-detect-F | | GGAACAGGTCCGGCGTGG |  |
| R2215-KO-detect-R | | AGTGGCCCGTACAGTGGC |  |
| R2462-KO-detect-F | | CCCTGGGCGACACCATCGG |  |
| R2462-KO-detect-R | | GGTCGCTGGTGTCGGTGG |  |
| R2468-KO-detect-F | | CGCGGGTCATGTCGAGCAC |  |
| R2468-KO-detect-R | | ATCAGCACCTTTACGCCGC |  |
| R2469-KO-detect-F | | TTGTCGGTCTCGGCCATGG |  |
| R2469-KO-detect-R | | AGATAGCCCTGCCCCAGG |  |
| R2507-KO-detect-F | | AGATGCACATGGCCGAGG |  |
| R2507-KO-detect-R | | CACCGGAGGCCTTCTGCG |  |
| R3283-KO-detect-F | | AACTGCATGGTGCCCTGGAG |  |
| R3283-KO-detect-R | | GTGCCATCGCTCTCTATGAGC |  |
| R3304-KO-detect-F | | GGGGTGTGGATCTGGGTCG |  |
| R3304-KO-detect-R | | GACCTCGCGCCGAGTACC |  |
| R3305-KO-detect-F | | CTTCGTAATGCGCCACCG |  |
| R3305-KO-detect-R | | AAGGTGAATGTGGCGGTACACG |  |
| R3698-KO-detect-F | | GGACCACCGAGCCGCTGAGG |  |
| R3698-KO-detect-R | | TACACCGCCTACGCCTACC |  |
| R3877-KO-detect-F | | GTCGTTTCAATCCATCGGCCTC |  |
| R3877-KO-detect-R | | GCGGCGTGTTCTCCATGCC |  |
| R3900-KO-detect-F | | TTCTGGTGGCCGGCGTGC |  |
| R3900-KO-detect-R | | CCTGGCGGTTCGGCGCGAG |  |
| R3981-KO-detect-F | | TAGTTGTTCCAGGCCCAGCC |  |
| R3981-KO-detect-R | | GGTTTTGCGCTCGGATTCG |  |
| R3994-KO-detect-F | | CGCCCAAGGACGTGATCC |  |
| R3994-KO-detect-R | | TCGGTGGCGATCAGGTCG |  |
| R4501-KO-detect-F | | CGACGGATAACGCCGACGC |  |
| R4501-KO-detect-R | | TGCACGCGGCCACCTTCC |  |
| pBBR1-HB-*rdmA*-F | | gtcgacggtatcgataagcttGGGACCCATGGGGAGAGAA | *Hind*III |
| pBBR1-HB-*rdmA*-R | | cgctctagaactagtggatccTCAATCGCGCATCACCAGC | *BamH*Ⅰ |
| pET32a-HB-*rdmA*-F | | gccatggctgatatcggatccCCCATGGGGAGAGAAAGCG | *Hind*III |
| pET32a-HB-*rdmA*-R | | ctcgagtgcggccgcaagcttTCAATCGCGCATCACCAGC | *BamH*Ⅰ |
| Inter*rdm*-*dmgA*-F | | GACTGGATGCCGACCGCGC |  |
| Inter*rdm*-*dmgA*-R | | CACGCCGGCCTTTTCGCAG |  |
| Inter*dmgA*-*B*-F | | AGCAACATCGGCGTGATGG |  |
| Inter*dmgA-B*-R | | GACCAGGTGATGGATCGGCC |  |
| Inter*dmgB-C*-F | | GCCTCCAGCGGCATGGAC |  |
| Inter*dmgB-C* -R | | TGGCGGTCACCAGGCAACG |  |
| Inter*dmgC-D* -F | | CACGCCAAGGAGGGCAAGG |  |
| Inter*dmgC-D*-R | | GGCGGCGACCACCGAGTAG |  |
| Inter*dmgD-E* -F | | GCCACCGAACAGTACATCCG |  |
| Inter*dmgD-E* -R | | GTGCCTGGGCTTCTTCCAGC |  |
| Inter*dmgE-F*-F | | GCCAATGCCGCCACCAAGG |  |
| Inter*dmgE-F*-R | | CTGCCCATCAGGTGGATGGC |  |
| Inter*dmgF-G*-F | | GCGCCTCTGGCTGTATGGC |  |
| Inter*dmgF-G* -R | | CGCTCTTGGCGGCGATGAC |  |
| Inter*dmgG-H*-F | | GCCTACGCCATCCTCTCCAGC |  |
| Inter*dmgG-H* -R | | GTTGGCCCAGCCGTTGAAC |  |
| *digA*-qPCR-F | | AATGGAGCACCAGTTCAA |  |
| *digA*-qPCR-R | | CACGAAGTTGACGATGAAG |  |
| *digB*-qPCR-F | | CGCTGTATCACATCTATGC |  |
| *digB*-qPCR-R | | CCACTTCTTCAGTTCCTTG |  |
| *digC*-qPCR-F | | TACCACATCATGGCGTTC |  |
| *digC*-qPCR-R | | CATCAGGGTGAAGGGATAG |  |
| *digD*-qPCR-F | | GAGTACGGCGAAGGTTAT |  |
| *digD*-qPCR-R | | AGGTGTAGTTGAGGGAGA |  |
| *rdmA*-qPCR-F | | ACTCGTAGACCAGCACTG |  |
| *rdmA*-qPCR-R | | ATCACTTCAAGAGCAAGGA |  |
| *dmgA*-qPCR-F | | CTGGAACAACTACGACAAG |  |
| *dmgA* -qPCR-R | | GCTTGGTAACGACGAATT |  |
| *dmgB*-qPCR-F | | GATCCATCACCTGGTCAA |  |
| *dmgB*-qPCR-R | | GCTCTGGTTGAACACTTC |  |
| *dmg*C-qPCR-F | | CGTCATCCAATCCGAACT |  |
| *dmgC*-qPCR-R | | CATCCAGTAGCAGGTTCA |  |
| *dmgD*-qPCR-F | | CCAGGAACACGAAGAACT |  |
| *dmgD*-qPCR-R | | CACCGAGTAGCTGTAGTC |  |
| *dmgE*-qPCR-F | | GAAACCCTTGACCAGGTC |  |
| *dmgE*-qPCR-R | | GTTCGGTTTGCGTTTCTG |  |
| *dmgF*-qPCR-F | | CAGAAGGTCATCGAGGAA |  |
| *dmgF*-qPCR-R | | TTCCAGGAAGTAGAAGGC |  |
| *dmgG*-qPCR-F | | CTACCTGAAGCAGAGCAA |  |
| *dmgG*-qPCR-R | | CTGATACCGTACTTCTTGAAC |  |
| *dmgH*-qPCR-F | | TATCGGCTTCACCAATGT |  |
| *dmgH*-qPCR-R | | GTCGCTGATCTCACTGAT |  |
| *rpoD*-qPCR-F | | GCATTCTCGAAGAGTACAAC |  |
| *rpoD*-qPCR-R | | GTCCTTGTCCTTATCCTTGG |  |
| P*_digA_*-F | | GAGGGTTCTCCAGGAGAGTGC |  |
| P*_digA_*-R | | GCCCACTCCTAAGCAACAGC |  |
| P*_digB_*-F | | CCTGCTTCTTGTTCGTCGATTC |  |
| P*_digB_*-R | | TTATTCTTGTCCTCGTACCTGAG |  |
| P*_digC_*-F | | GTGGGTGAAGGCGCGCAG |  |
| P*_digC_*-R | | CGACTTCTCCGTACTTTTTCTC |  |
| P*_digD_*-F | | CTCCACGCCGTCGCGCTC |  |
| P*_digD_*-R | | GTGGACACCCTGTGGCTGAT |  |
| P*_dmgA_*-F | | GCTTTCTCTCCCCATGGGTCC |  |
| P*_dmgA_*-R | | TGTTCTGCCCGTGACGCTG |  |
| P*_dmgA_*_-F2_-F | | GCTTTCTCTCCCCATGGGTCC |  |
| P*_dmgA_*_-F2_-R | | AGCAAGCGCTTGGTTGAAAAGC |  |
| P*_dmgA_*_-F3_-F | | GCTTTCTCTCCCCATGGGTCC |  |
| P*_dmgA_*_-F3_-R | | CCAAGCGCTTGCTTGGTTGG |  |
| P*_dmgA_*_-F4_-F | | GCTTTCTCTCCCCATGGGTCC |  |
| P*_dmgA_*_-F4_-R | | TGGTTGGCACTCGCGGGC |  |

*^a^*The underlined nucleotide sequences are digestion sites of restriction endonuclease.

PCR = polymerase chain reaction.

P*_dmgA_*= the promoter region of gene *dmgA*.

Inter*dmgA*-*B*= the intergenic region of gene *dmgA*-*dmgB*.
