# Supplementary material for: RdmA Is a Key Regulator in Autoinduction of DSF Quorum Quenching in Pseudomonas nitroreducens HS-18
Source: mBio. 2022 Dec 20;14(1):e03010-22. doi: 10.1128/mbio.03010-22 (PMC9973270; doi:10.1128/mbio.03010-22)
Supplement: TABLE S4 [file mbio.03010-22-s0009.docx]

**Table S4** The chemical structures of DSF and fatty acid derivatives

| Molecular name | Structure | | | Formula | | |
| --- | --- | --- | --- | --- | --- | --- |
| DSF(*cis*-11-methyldodecenoic- acid) |  | | | C_13_H_24_O_2_ | | |
| PDSF(*cis*-2-decenoic acid) |  | | | C_10_H_18_O_2_ | | |
| BDSF(*cis*-2-dodecenoic acid) |  | | | C_12_H_22_O_2_ | | |
|  |  | | |  | | |
| Lauric acid |  | | | C_12_H_24_O_2_ | | |
| Myristic acid |  | | | C_14_H_26_O_2_ | | |
| Palmitic acid |  | | | C_16_H_32_O_2_ | | |
| Linoleic acid |  | | C_18_H_32_O_2_ | | |  |
| Oleic acid |  | C_18_H_34_O_2_ | | |  |  |
| Erucic acid |  | C_22_H_42_O_2_ | | |  |  |
| Hexadecane |  | C_16_H_32_ | | |  |  |
| Citronellol |  | C_10_H_20_O | | |  |  |
